# A rotenone organotypic whole hemisphere slice model of mitochondrial abnormalities in the neonatal brain

**DOI:** 10.1186/s13036-024-00465-w

**Published:** 2024-11-14

**Authors:** Brendan Butler, Malcolm Renney, Kristin Bennett, Gisele Charpentier, Elizabeth Nance

**Affiliations:** 1 Department of Chemical Engineering, University of Washington, Seattle, WA 98195 USA; 2 Department of Bioengineering, University of Washington, Seattle, WA 98195 USA; 3 Molecular Engineering & Sciences Institute, University of Washington, Seattle, WA 98195 USA

**Keywords:** Ex vivo model, Neurodevelopment, Cellular metabolism, Confocal microscopy, Nanoparticle tracking

## Abstract

Mitochondrial abnormalities underscore a variety of neurologic injuries and diseases and are well-studied in adult populations. Clinical studies identify critical roles of mitochondria in a wide range of developmental brain injuries, but models that capture mitochondrial abnormalities in systems representative of the neonatal brain environment are lacking. Here, we develop an organotypic whole-hemisphere (OWH) brain slice model of mitochondrial dysfunction in the neonatal brain. We extended the utility of complex I inhibitor rotenone (ROT), canonically used in models of adult neurodegenerative diseases, to inflict mitochondrial damage in OWH slices from term-equivalent rats. We quantified whole-slice health over 6 days of exposure for a range of doses represented in ROT literature. We identified 50 nM ROT as a suitable exposure level for OWH slices to inflict injury without compromising viability. At the selected exposure level, we confirmed exposure- and time-dependent mitochondrial responses showing differences in mitochondrial fluorescence and nuclear localization using MitoTracker imaging in live OWH slices and dysregulated mitochondrial markers via RT-qPCR screening. We leveraged the regional structures present in OWH slices to quantify cell density and cell death in the cortex and the midbrain regions, observing higher susceptibilities to damage in the midbrain as a function of exposure and culture time. We supplemented these findings with analysis of microglia and mature neurons showing time-, region-, and exposure-dependent differences in microglial responses. We demonstrated changes in tissue microstructure as a function of region, culture time, and exposure level using live-video epifluorescence microscopy of extracellularly diffusing nanoparticle probes in live OWH slices. Our results highlight severity-, time-, and region-dependent responses and establish a complimentary model system of mitochondrial abnormalities for high-throughput or live-tissue experimental needs.

**Supplementary Information:**

The online version contains supplementary material available at 10.1186/s13036-024-00465-w.

## Introduction

In the brain, mitochondria play critical roles in processes that dictate cell function, communication, and fate [1]. Mitochondria are critical to energy generation and involved in intercellular calcium clearance, lipid biogenesis, radical oxygen species (ROS) regulation, and cell communication [1]. Collectively, all neural cells work intimately to maintain a tightly regulated neurometabolic unit that is responsible for 20% of the body’s total energy utilization, which is primarily derived from mitochondrial oxidative phosphorylation [2–5]. The repertoire of roles mitochondria have in neural cells and the brain’s reliance on mitochondrial energy links metabolic dysfunctions to a variety of neurologic conditions, including stress, aging, neurodegeneration, traumatic brain injury, post-traumatic stress disorder, stroke, and neuropsychiatric disorders [6–14].

While mitochondria are well-studied in adult neurologic injury and disease, less is known about their roles in developmental brain injury, where the energy landscape is vastly different [15]. The size of the developing brain ranges between one-fourth and one-third that of the adult brain and is estimated to utilize nearly twice as much glucose as the adult brain for growth, maturation, synaptic pruning, and restructuring [16, 17]. Regional metabolic activity profiles in the developing brain are also complex, temporally nonlinear and varying with gestational and developmental age [18]. At the mitochondrial level, differences in respiratory capacity, electron transport chain protein expression, and recovery of metabolism post-injury are shown to depend on sex and developmental age [19–23]. The heightened energy demands of neurodevelopment render the developing brain more sensitive to metabolic disruptions. Moreover, there are several unique avenues by which neonatal populations experience mitochondrial abnormalities. Clinical studies have connected preterm birth, placental lesions, intrauterine growth restriction, intermittent hypoxia, maternal stress, pain, childhood trauma, exposure to pollutants from fine particulates (PM_2.5_), and glutamate excitotoxicity with differences in mitochondrial respiratory capacity, density, gene expression, protein expression, and ROS levels [6]. Differentials in mitochondrial dysfunction levels were also reported when accounting for biological sex and maternal ethnicity [6]. Other avenues for mitochondrial abnormality in the neonatal brain include early-life stress, iron and thyroid deficiencies, and exposure to morphine and caffeine [24–28].

Despite the variety of circumstances in which neonatal populations can experience mitochondrial abnormalities, the multi-scalar effects of mitochondrial dysfunction on the developing brain remain largely unexplored and many studies of mitochondrial abnormalities as outcomes of specific injuries and disease states focus on specific brain regions or individual cell types [29–39]. Here, we leverage an organotypic whole-hemisphere (OWH) brain slice platform to model mitochondria-driven dysfunction in the neonatal brain. OWH slices reorganize in structure throughout culture and in response to biological stimuli, providing a dynamic system to study the brain environment [40–44]. Moreover, OWH slices provide a balance between experimental throughput and native representation of the brain environment and limit animal-to-animal variability, with the opportunity to generate on average 20 OWH slices per hemisphere. We have demonstrated that OWH slices obtained from postnatal (P) day 10 rats, term equivalent to the human neonate, retain viability and metabolic activity up to two weeks in vitro [44]. This postnatal age and culturing window are well-suited to study healthy and abnormal mitochondrial dynamics in the neonatal brain.

To systemically drive mitochondrial abnormality, we expose OWH slices to low-grade doses of mitochondrial toxin rotenone (ROT), a potent inhibitor of respiratory chain protein Complex I, canonically used to study mitochondrial dysfunction in models of neurodegeneration [45–47]. We use ROT to inflict mitochondrial damage and examine the tissue-level, extracellular, cell-type, organelle, and RNA-level responses to repeat ROT exposure and single ROT exposure in two distinct brain regions over 6 days in culture. Our results highlight severity-, time-, and region-dependent responses and establish ROT exposure in term-equivalent OWH cultures as a model system complimentary to traditional in vivo or in vitro ROT models.

## Methods

### Animal work and ethics statement

All animal work was performed in accordance with the recommendations in the Guide for the Care and Use of Laboratory Animals of the National Institutes of Health (NIH). Animals were handled according to approved Institutional Animal Care and Use Committee (IACUC) protocol (#4383–02) of the University of Washington, Seattle, WA, USA. The University of Washington has an approved Animal Welfare Assurance (#A3464–01) on file with the NIH Office of Laboratory Animal Welfare (OLAW), is registered with the United States Department of Agriculture (USDA, certificate #91-R-0001), and is accredited by AAALAC International. Every effort was made to minimize suffering. All work was performed using Sprague–Dawley (SD) rats (virus antibody-free, Rattus norvegicus, IGS, Charles River Laboratories, Raleigh, NC, USA) that arrived at postnatal (P) day 5 with a nursing dam. Before removal from the dam at P10, each dam and her pups were housed under standard conditions with an automatic 12 h light/dark cycle, temperature range of 20–26 °C, and access to standard chow and sterile tap water ad libitum. The pups were checked for health daily. All animal studies for this work were routinely started in the mornings to eliminate any influence of time of day. To eliminate the influence of sex-based differences, all animals used were female.

### Preparation of coronal OWH brain slice cultures

Healthy female SD P10-P11 rats were injected with an overdose of 100μL pentobarbital (Commercial Beuthanasia D, 390 mg/mL pentobarbital, administered > 120–150 mg/ kg) intraperitoneally. Once the animal was unresponsive to a toe pinch with tweezers, it was decapitated with surgical scissors. The brain was removed rapidly under aseptic conditions and submerged in ice cold dissection media consisting of 100% HBSS (Hank’s Balanced Salt Solution, no Mg^2+^, no Ca^2+^), Thermo Fisher Scientific, Waltham, MA, USA), 1% Penicillin–Streptomycin (Thermo Fisher Scientific), and 0.64% w/v glucose (MilliporeSigma, Burlington, MA, USA). Whole brains were split into hemispheres with a sterile razor blade and sliced coronally into 300 μm-thick sections with a Mcllwain tissue chopper (Ted Pella, Inc., Redding, CA, USA). Individual slices were separated in ice cold dissection media using sterile fine tip paint brushes and transferred onto 30-mm 0.4-μm-pore-sized cell culture inserts (hydrophilic PET, CellTreat Scientific Products, Pepperell, MA) before being placed in a non-treated 6-well plate (USA Scientific Inc., Ocala, FL, USA) containing 1 mL pre-warmed (37 °C) slice culture media (SCM; 50% MEM [minimum essential medium, no glutamine, no phenol red, Thermo Fisher Scientific], 41.75% HBSS [with Mg^2+^, with Ca^2+^], 5% horse serum [heat inactivated, New Zealand origin, Thermo Fisher Scientific], 1.25% HEPES [Thermo Fisher Scientific], 0.575% w/v glucose, 1% GlutaMAX Supplement, and 1% Penicillin–Streptomycin). Slices were cultured in a sterile CO_2_ incubator (Thermo Fisher Scientific) at 37 °C with constant humidity, 95% air and 5% CO_2_. Proper aseptic techniques and standard checks that are published for maintenance of OWH slice cultures were used to avoid contamination through 10 days in culture [44, 48]. Media was routinely checked for cloudiness and slices were visually checked daily for changes in transparency, discoloration, swelling, or volume loss. Slice samples that did not meet quality criteria were discarded from studies.

### ROT exposure in OWH slices

**Fig. 1:**
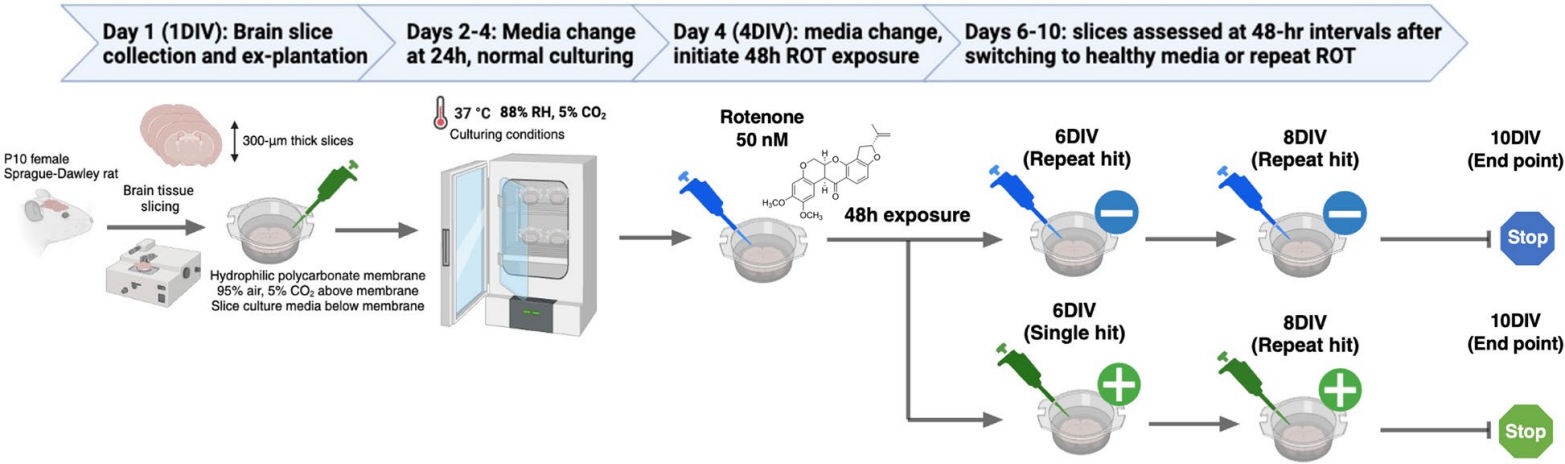
Schematic of OWH slice preparation and ROT experimental workflow. 300-μm thick coronal OWH slices are prepared from P10 female rats. Slices are plated on polycarbonate membrane inserts above culture media. Media is changed < 30 min after ex-plantation onto membrane inserts and at 24 h (2DIV) before 48 h of ROT exposure (red pipette). Every 48 h starting at 6DIV, slices are given healthy media (green) or repeat hits of ROT (blue) through 10DIV (end point for single- and repeat-hit exposures)

ROT (MW = 394.4 mg/mmol) is initially dissolved in 250 μL dimethylsulfoxide (DMSO) which we define as RDMSO (DMSO containing ROT) at 39.44 mg/mL for a stock concentration of 100 mM. Because the solubility and stability of ROT in aqueous media is limited, stock RDMSO was further diluted in DMSO to 1 mM RDMSO and stored in 15 μL aliquots at—20 °C before further dilution into SCM to make ROT-doped SCM (RSCM, defined as SCM containing ROT). For each exposure, RSCM was always made fresh without exposure to light and limited freeze–thaw cycles. RDMSO aliquots (1 mM) were thawed and diluted into pre-warmed 37 °C SCM at 1:100 to achieve 10 μM RSCM, and further diluted 1:200 for 50 nM RSCM. RSCM solutions were inverted to mix without vortexing. All slices are obtained from P10 female rats; all slices are cultured 4 days to restore viability after tissue ex-plantation. At 4 days in vitro (4DIV), SCM is replaced with RSCM at 50 nM (Fig. 1). For the repeat-hit exposure, slices are incubated with RSCM for 2 days and replaced with RSCM at 2-day increments out to 10DIV. For the single-hit exposure, slices are incubated with RSCM for 2 days and replaced with healthy media at 2-day increments out to 10DIV. Our healthy control slices are slices cultured without ROT, with media changes at identical time points.

### Lactate dehydrogenase (LDH) assay for whole-slice cytotoxicity

To measure whole-slice cytotoxicity in response to ROT exposure, one brain slice was plated per insert, with 3 total slices per treatment condition. Culture supernatants were collected acutely, at 4DIV, then every 2 days out to 10DIV. At 4DIV, slice media was replaced with 1 nM, 50 nM, 100 nM, or 10 μΜ RSCM. Supernatants were collected and repeatedly replaced with RSCM every 48 h, which we designate as a repeat-exposure regime with the suffix ‘-R’ following the ROT concentration. All supernatant samples were immediately stored at − 80 °C. Supernatant samples were removed and thawed at room temperature to conduct LDH assays (601,170, Cayman Chemical, Ann Arbor, MI, USA). Following the manufacturer’s instructions, 100 μL of LDH reaction buffer was added to 100 μL of sample supernatant in triplicate to 96-well plates on ice, and the plates were transferred to a stir plate in a 37 °C incubator. After 30 min, absorbance at 490 nm was measured on a Synergy H1 multimode microplate reader to detect the production of colorimetric formazan. Background absorbance readings of 200 μL SCM (no LDH reagent added) were subtracted from all absorbance values.

### Propidium iodide (PI) staining for % cell damage

To measure regional cell death, slices were stained with 1 mL of 5 μg/mL PI (Thermo Fisher Scientific) in SCM for 45 min at standard culture conditions. At the conclusion of each ROT exposure media, ROT media was inmmediately removed and placed with PI-SCM staining solution. The staining solution was placed underneath the insert. Slices were washed twice for 3 min each with sterile 1xPBS at room temperature, followed by a 1 h wash with 37 °C SCM at culturing conditions, and then formalin fixed for 1 h with 10% formalin phosphate buffer (Thermo Fisher Scientific). Following two final washes with room temperature 1xPBS, slices were stored covered at 4 °C until ready for use. Within 1 week, slices were stained for 30 min with 0.1 μg/mL 4’,6- diamindino-2-phylindole (DAPI; Thermo Fisher Scientific) in 1xPBS at room temperature. Slices were washed twice for 3 min each with 1xPBS prior to imaging. Two-channel 40 × confocal images (oil immersion, 1.30 numerical aperture, Nikon Corporation, Minato City, Tokyo, Japan) were obtained for PI and DAPI. For every slice, 5–10 images were acquired from both the cortex and midbrain. Image acquisition settings were consistent for all images and conditions. For each image, DAPI + cell nuclei (total cells) and PI + cell nuclei (dead cells) that were also DAPI + were counted manually in ImageJ (NIH) after applying an Otsu threshold and fluorescent cutoff to aid in visualization consistent with image processing methods in prior work [43, 44, 48–50]. The background fluorescent cutoff was kept consistent across all images. The PI + /DAPI + cell ratio was expressed as the percentage of dead cells in an individual image.

### Live-slice MitoTracker staining and complex I imaging

To compare mitochondrial responses across healthy and ROT-exposed OWH slices, we leveraged MitoTracker (MT) DeepRed (ThermoFisher M22426, Waltham, MA), a fluorescent probe which accumulates in mitochondria in a membrane-potential dependent manner when staining live tissue. At the conclusion of each time point, live brain slices from P10 female rats were stained with MitoTracker. First, 5 drops of Hoescht NucBlue live nuclear stain are added to 1 mL pre-warmed 37 °C SCM. To add the MitoTracker stain, 1 mM MitoTracker stock solution in DMSO, MitoTracker was diluted to a working solution of 500 nM in pre-warmed 37 °C Hoescht-doped SCM (0.5 μL MitoTracker in 999.5 μL SCM). 1 mL Hoescht-MitoTracker-SCM was added below the membrane insert and 100 μL was applied on top of each brain slice followed by a 1 h incubation at 37 °C with constant humidity, 95% air, and 5% CO2. Slices were washed once with warmed SCM for 3 min, and then immediately fixed in formalin for 1 h. Following fixation, we washed slices twice in 1 × PBS for 3 min. Next, we co-stained the OWH slices with a complex I antibody (Abcam EPR11521B, Cambridge, MA) at 1:250 dilution in PBS supplemented with 1% Triton X-100 and 3% goat serum overnight at 4 °C. The next day, the OWH slices were washed twice with 1 × PBS for 3 min each time, then were incubated in an AlexaFluor 488 goat anti-rabbit (ThermoFisher Scientific Cat # A11034) secondary antibody solution at 1:500 dilution for 2 h at room temperature in PBS with 1% Triton X-100 before DAPI staining. For confocal imaging, a 10 × tilescan of each slice was obtained to provide a representative region map, followed by additional images at 240x (60 × lens with 4 × zoom) for representative mitochondrial morphology and complex I association. All slice imaging was completed within 1 week of fixation.

### Reverse-transcriptase quantitative polymerase chain reaction (RT-qPCR)

RNA was extracted from OWH slices for RT-qPCR, using the protocol adapted from the methods used by Nguyen et al. OWH slices (3 per insert) were cultured on the same membrane with SCM as described [50]. At each specified time point, slices were removed from inserts and were immediately preserved in RNALater (Thermofisher, Waltham, MA, USA) and kept at 4 °C prior to processing to prevent RNA degradation. The RNA from homogenized brain slices were extracted with TRIzol reagent, pelleted at 15,000 × g, washed several times with ultrapure diethylpyrocarbonate (DEPC)-treated water and 70% ethanol, and the RNA final concentration was measured using a NanoDrop. cDNA was diluted to 20 ng/μL with ultrapure RNA-free water. RNA was transcribed into cDNA using Thermofisher (Waltham, MA, USA) Reverse Transcription RNA to cDNA kit. qPCR was run using the transcribed cDNA and BioRad (Hercules, CA, USA) SYBR Green Master Mix that binds to double-stranded DNA to quantitatively track the progress of DNA amplification in real-time. We examined expression of complex I encoding subunit NDUFS-1, SIRT-6 for mitochondrial regulation, MFF-1 and MFN-1 for fission and fusion, BCL-2 and AIFM-1 as mitochondrial apoptotic process markers, and MMP-9 as a probe for protease-driven remodeling of the extracellular matrix (ECM) (Table 1). Tom20 (mitochondrial membrane protein) was used as a reference housekeeping gene. The qPCR runs at 95° for 30 s, 95 °C for 5 s, and then 60 °C for 30 s for 40 cycles.

**Table 1:** Genes and associated primers screened with RT-qPCR in ROT-exposed slices

Role	Name	Description	NCBI reference	Sequence
Housekeeping	Tom20	Housekeeping gene, mitochondrial biomass	NM_152935.2	F-ACTCCCATTCTTCCACCTTTG R-CCCTGTTGCTGTAGCCATATT
Mitochondrial gene regulation	SIRT-6	Nuclear genes for mitochondrial products	NM_001031649.1	F-TGCCAGCAAGGTTCTTACTAC R-GATGATCTCCTGTGCGACTTT
	MFN-1	Mitochondrial fusion transcription factor	NM_138976.2	F-TCCTCTTCCTCAGTGCTAGTT R-CAGACCGTCCACTTCACATTAG
	MFF-1	Mitochondrial elongation factor 1	NM_130894.4	F-GACAAAGGTGCCTTCAGTAGAT R-CACGGTTCCGGTAGTAAGAAAG
Apoptosis	Bcl-2	Mitochondrial apoptosis regulator	NM_017059.2	F-AGGCGAATTGGCGATGAA R-CTTCTTCCAGATGGTGAGTGAG
	Aifm1	Mitochondria-associated apoptosis inducing factor	NM_031356.2	F-CATACATGCGACCTCCTCTTT R-CACTCCCTCTCGTTTGACTTT
ECM remodeling	MMP-9	Matrix metalloproteinase 9	NM_031055.2	F- CGCCAACTATGACCAGGATAAG R- GTTTAGAGCCACGACCATACAG

### Immunofluorescence staining and confocal microscopy of mature neurons and microglia in formalin-fixed OWH slices

OWH slices were prepared, stained with PI, and formalin fixed in the same manner they were for PI staining and imaging studies. Following fixation, OWH slices were incubated with primary antibodies for Iba1 (microglia) or NeuN (neurons). For microglia staining, OWH slices were incubated overnight (16 h) at 4 °C in anti-Iba1 (rabbit anti-Iba, Wako Cat #019–19,741) at 1:250 dilution in 1 × PBS containing 1% Triton X-100 and 3% goat serum. The next day, the OWH slices were washed twice with 1 × PBS for 3 min each time, then were incubated in an AlexaFluor 488 goat anti-rabbit (ThermoFisher Scientific Cat # A11034) secondary antibody solution at 1:500 dilution for 2 h at room temperature in the same buffer conditions as the primary antibody. For mature neuron staining, OWH slices were incubated overnight at 4 °C in anti-NeuN (rabbit anti- NeuN) at 1:500 dilution in 1 × PBS containing 1% Triton X-100 and 3% goat serum and washed as described for NeuN. Following two 3-min washes with 1 × PBS wash, cellular nuclei were stained with a 0.1 μg/mL solution of DAPI in 1 × PBS for 30 min at room temperature. Following two 3-min 1 × PBS washes, OWH slices were stored at 4 °C until they were imaged. Two-channel 40 × confocal images (oil immersion, 1.30 numerical aperture, Nikon Corporation, Minato City, Tokyo, Japan) were obtained. For every slice, 5–10 images were acquired from both the cortex and midbrain. Image acquisition settings were consistent for all images and conditions. Separate ImageJ macros were run to determine the localization of the AF488 somatic markers with PI + and DAPI + nuclei, keeping identical Otsu thresholding settings used to quantify PI/DAPI ratios, as well as a 20-pixel size cutoff. The degree of PI + co-localization with the individual cell stains was quantified by dividing the number PI + nuclei associated with the AF488 stain by the number of DAPI + nuclei associated with the AF488 stain.

### Nanoparticle preparation and characterization

40 nm fluorescent carboxylate (COOH)-modified polystyrene latex (PS) nanoparticles (PS-COOH) (Thermo Fisher Scientific) were covalently modified with methoxy (MeO)- poly(ethylene glycol) (PEG)- amine (NH_2_) (5 kDa MW, Creative PEGWorks, Durham, NC, USA) by carboxyl amine reaction as previously described and with no changes to prior protocol [51]. The hydrodynamic diameter and polydispersity index (PDI) of the resulting PEG- conjugated fluorescent nanoparticles were measured via dynamic light scattering (DLS) and the ζ-potential by laser Doppler anemometry. Both DLS and laser Doppler anemometry were performed using the Zetasizer Nano ZS (Malvern Panalytical, Malvern, UK). Particles were diluted to ~ 0.002% solids in filtered (0.45 µm, Whatman, Maidstone, UK) 10 mM NaCl, pH 7.0, prior to measurement.

### Multiple-particle tracking (MPT) and analysis in live ex vivo slices

For MPT experiments in live brain slices, one slice was plated per insert (*N* = 2–3 slices per experimental condition). Particles were tracked in ROT-exposed slices immediately after each exposure window, with all tracking completed within 2 h of the end of the exposure window, including particle incubation time. At the specified timepoints in Fig. 1, SCM was exchanged for 1 mL prewarmed (37 °C) SCM containing 40 nm PS-PEG nanoparticles (100 μg/mL) and Hoechst (5 drops/mL, NucBlue Live ReadyProbes Reagent, Hoechst 33,342, ThermoFisher Scientific). 900μL of nanoparticle-Hoechst-SCM solution was added below the insert and 100 μL was added dropwise to the top of the slice. OWH slices were incubated for 1 h at standard culture conditions then washed once with 1 mL warm (37 °C) SCM for 3 min each. Following the wash, OWH slices were transferred to an imaging dish and kept in a temperature-controlled incubation chamber maintained at 37 °C, 5% CO_2_, and 88% relative humidity during the imaging session. All video acquisition was completed within 1 h. Three videos were collected and quantified from the cortex and midbrain of each slice. Videos were collected at 67 frames-per-second, and 100 × magnification (oil immersion, 1.45 numerical aperture, Nikon Corporation) for 651 frames via fluorescent microscopy using a cMOS camera (Hamamatsu Photonics, Hamamatsu City, Japan) mounted on a confocal microscope. Nanoparticle trajectories, trajectory MSDs, and *D*_*b,eff*_ values were extracted from microscopy videos via a lab-developed Python package diff_classifier for parallelized and reproducible MPT workflows as previously described with no change in protocol [44, 52, 53].

### Statistical analysis

All statistical analyses were carried out in GraphPad Prism (GraphPad Software Inc). For all tests run, differences were defined as statistically significant at *p* < 0.05, which corresponds to a single asterisk (*) on significance brackets in all figures. Multiple asterisks denote significance values below 0.05, where two asterisks refer to *p* < 0.01, three asterisks refer to *p* < 0.001, and four asterisks refer to *p* < 0.0001. The significance cutoffs apply to statistics in all figures. The D’Agostino-Pearson omnibus K2 test was first used to test for normality for all datasets (normality QQ plots are provided in Fig. S1-S3). If we were unable to reject the null hypothesis that data were sampled from a population that follows a Gaussian distribution, we either ran an ordinary one-way ANOVA or Brown-Forsythe and Welch ANOVA test, depending on if we could assume equal standard deviations. If we were able to reject the null hypothesis that the data were taken from a normally distributed population, we used the Kruskal–Wallis test for significance. In these instances, we applied Dunn’s method to correct for multiple comparisons.

## Results

### Culture time drives dose-dependence of the extent of ROT-induced injury in OWH brain slices

**Fig. 2:**
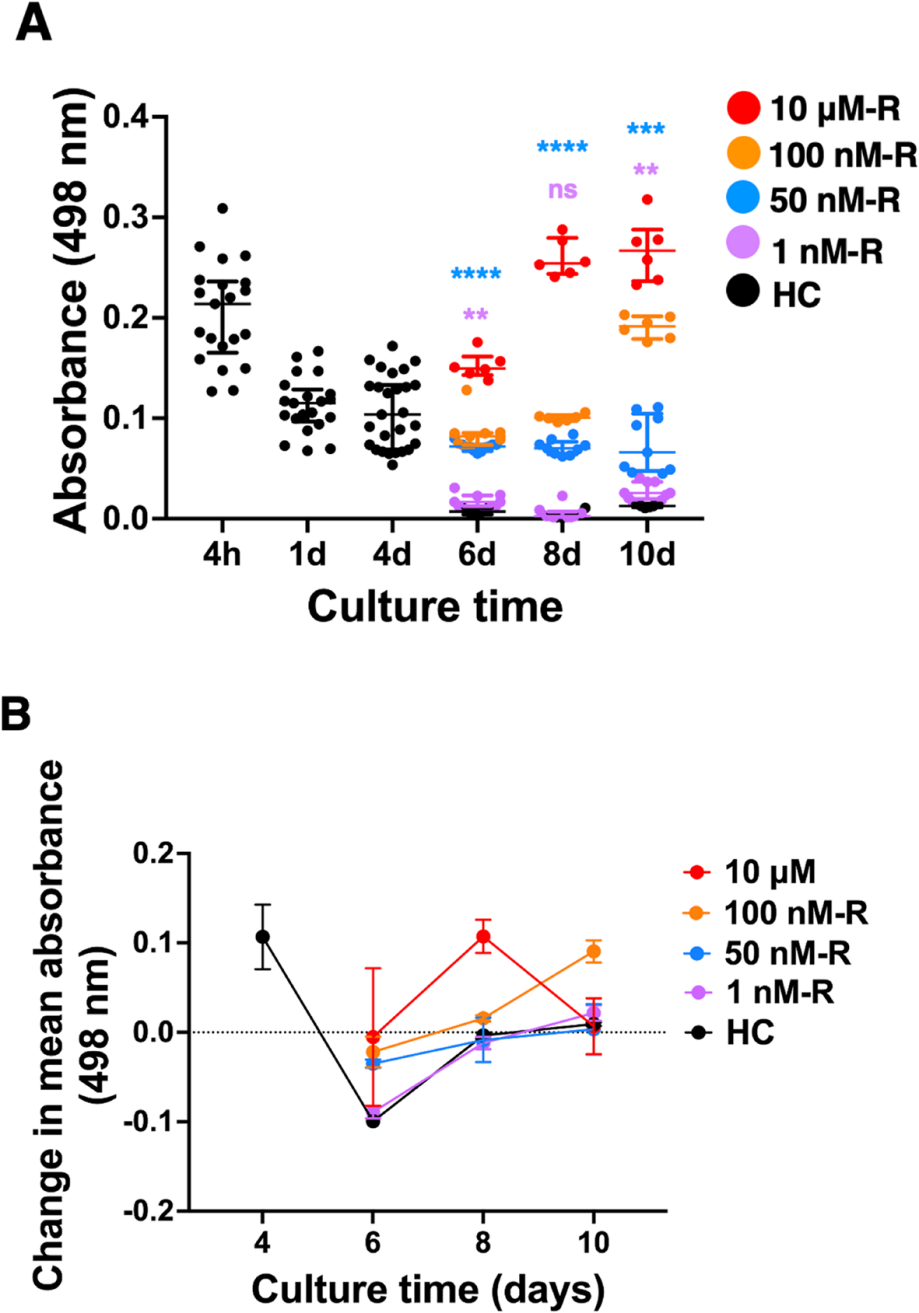
Overall OWH slice health in response to ROT measured by LDH absorbance. **A** Whole-slice cytotoxicity screening experiment for lactate dehydrogenase (LDH) concentration in supernatants from OWH slices exposed to 1 nM-R through 10 μΜ-R ROT through 10DIV. Color-coded significance indicators represent ANOVA results for respective ROT concentrations compared against healthy OWH slices from 6DIV – 10DIV. **p < 0.01, ***p < 0.001, ****p < 0.0001. **B** Differential LDH absorbance by exposure level from 4DIV – 10DIV. Data points represent the difference in mean absorbance between time points for each exposure group. Each data point represents a technical replicate (*n* = 3) from three to six separate OWH slices (*N* = 3–6)

Typically, ROT-exposure models involve multiple low-grade exposures over days to weeks to study chronic effects of mitochondrial abnormalities. To first determine if we could sustain ROT-driven injury in our term eqiuvalent OWH slices over longer times without compromising overall OWH slice health, we measured LDH released from OWH slices during 6 days of ROT exposure, starting at 4DIV. Based on evidence that nanomolar concentrations (> 20 nM) are reported to inhibit complex I activity in vitro, exposures for in vitro models range from 1-200 nM, and upper-limit in vitro cytotoxicity levels are reported between 1–20 μΜ in various models [29, 32, 34, 41, 45, 54–61], we selected four concentrations to screen overall OWH slice health following repeated exposure (R): 1 nM-R, 50 nM-R, 100 nΜ-R, and 10 μM-R. From 6DIV through 10DIV, one-way ANOVA of concentration showed that 10-μΜ-R was the only group to result in significant injury independent of culture time (*p* < 0.05). To demonstrate the effects of ROT concentration and culture time, we performed a two-way ANOVA to show that the effects of culture time, ROT concentration, and time-concentration interaction are all significant (*p* < 0.0001). After 48 h of exposure (6DIV), all concentrations resulted in significantly higher LDH release compared to healthy control levels (*p* < 0.0001), with the 1 nM-R injury less significant (*p* < 0.01). At 8DIV, a similar trend was observed where every concentration except 1 nM-R resulted in significantly higher LDH release compared to healthy OWH slices (*p* < 0.0001) (Fig. 2A). The 1 nM-R group showed significant injury by 10DIV (*p* < 0.01) but remained closest in value to healthy control levels. By 10DIV, all other ROT concentrations resulted in significantly higher LDH release compared to healthy controls, with 50 nM-R resulting in the most consistent injury relative to healthy OWH slices over the culturing window.

To better visualize the effect of culture time on the injury profile, we plotted mean differential LDH absorbance between each DIV starting at 4DIV (Fig. 2B). We observed that differential LDH release from 1 nM-R and 50 nM-R groups paralleled HC LDH release through 10DIV, but the 100 nM-R and 10 μΜ-R differential LDH release profiles diverged at 8DIV. Additionally, 8DIV represented an inflection point after which the change in absorbance was positive. The 50 nM-R exposure level resulted in injury relative to healthy OWH slices with minimal temporal dependence, compared to 100 nM-R and 10 μΜ exposures. We concluded that 50 nM ROT is an ideal candidate to drive injury in OWH slices through 10DIV, with an inflection response in the whole-slice response occurring at 8DIV. We also moved forward with a 10 μM ROT exposure to be used as a positive control or more significant cell death.

### ROT exposure alters mitochondrial responses in OWH slices in a regime-dependent manner

**Fig. 3:**
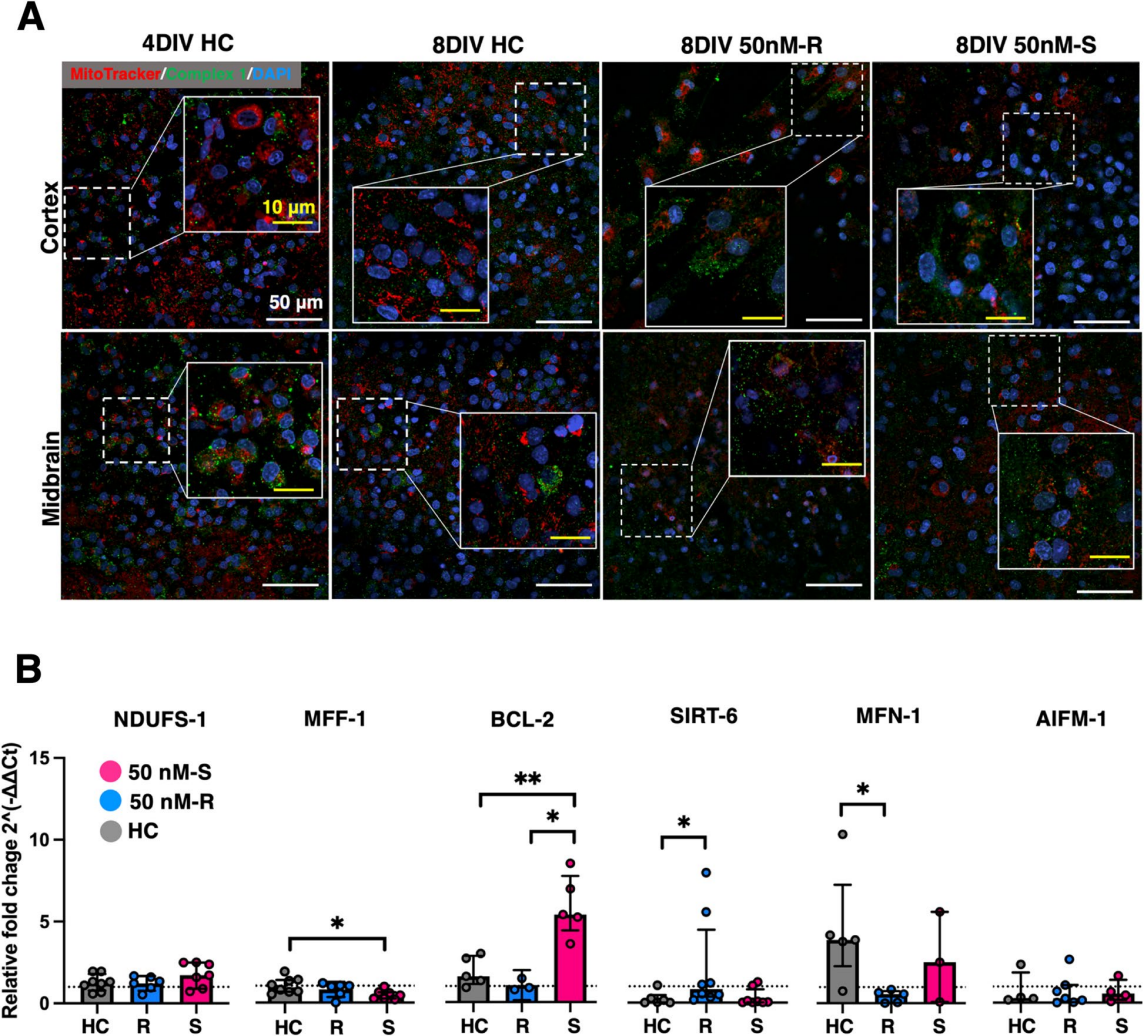
Mitochondrial responses during ROT exposure. **A** Representative images of live mitochondria (red) in the midbrain of healthy and ROT (50 nM, 10 μΜ) exposed slices (*N* = 1–2 OWH slices per group, 5–10 representative images taken at 240 × magnification in the midbrain). Cell nuclei (blue) are stained with DAPI. Scale bars: 10 µm in all images. **B** 8DIV fold change expression relative 4DIV HC samples for complex I, mitochondrial gene regulatory markers, morphology markers, and mitochondrial apoptosis regulatory markers for healthy OWH slices (HC), repeat-exposed 50 nM (50 nM-R), and single-exposed 50 nM (50 nM-S). *p < 0.05 and **p < 0.01

After identifying a sufficient ROT exposure level to cause injury but not kill the OWH slice, we qualitatively analyzed mitochondrial morphology, fluorescence-dependent membrane potential, and spatial orientation in ROT-exposed OWH slices at the 8DIV inflection point in whole-slice health following 50 nM-R exposure. We hypothesized that repeated exposure to the toxin would result in more morphological and fluorescence shifts from bright punctae to less-fluorescent, dispersed objects with more diffuse signal, as well as less cellular localization of the mitochondria. We did not expect ROT exposure to interfere with the fluorescence of the complex I signal due to the ROT binding site being within the mitochondrial inner membrane, but we did expect more extracellular distribution of complex I signal following changes to mitochondrial integrity due to cell death. Additionally, following the results in Fig. 2 and evidence that ROT-driven injury is dose- and time-dependent [54], we incorporated a single-exposure group (S) to compare mitochondrial responses and injury patterns as a function of the number of exposures to the toxin. The fluorescence intensity of complex I did not appear affected by the vehicle but showed more extracellular presence (Fig. 3A), likely as a result of cell death. Regional differences were most pronounced following repeat exposure in ROT-exposed slices. OWH slices given 50 nM-S exposure showed mitochondrial populations that more resembled healthy OWH slices, evidenced by quantity of nuclei, but with a more heterogeneous distribution of mitochondrial fluorescence.

We used RT-qPCR to quantify fold changes in expression of genes encoding for complex I (NDUFS-1), mitochondrial fission and fusion (MFF-1, MFN-1), mitochondrial regulatory marker SIRT-6, and mitochondrial-activated apoptosis (BCL-2, AIFM-1). Expression was calculated relative to mitochondrial membrane protein Tom20 as a housekeeping gene to control for anticipated changes in mitochondrial biomass during culturing. Complex I expression aligned with images and showed consistent signal independent of ROT exposure. 50 nM-R resulted in comparable expression relative to healthy OWH slices (Fig. 3B, Table 1). While the results were not statistically significant (*p* = 0.322), 50 nM-S resulted in an elevated expression of complex I, which may reflect a compensatory response. We observed lesser expression of fission marker MFF-1 in 50 nM-S OWH slices relative to healthy controls (*p* < 0.05) and upregulation of fusion marker MFN-1 in 50 nM-R OWH slices (*p* < 0.05) which might suggest a shift away from pro-fission states. Repeat exposure did not result in less expression of BCL-2, but 50 nM-S resulted in significantly higher expression compared to the HC (*p* < 0.01) and 50 nM-R (*p* < 0.05), which could indicate that the degree of exposure influences the ability to regulate apoptotic markers.

### DAPI and propidium iodide (PI) staining captures time-dependent regional changes in cell density and cytotoxicity

**Fig. 4:**
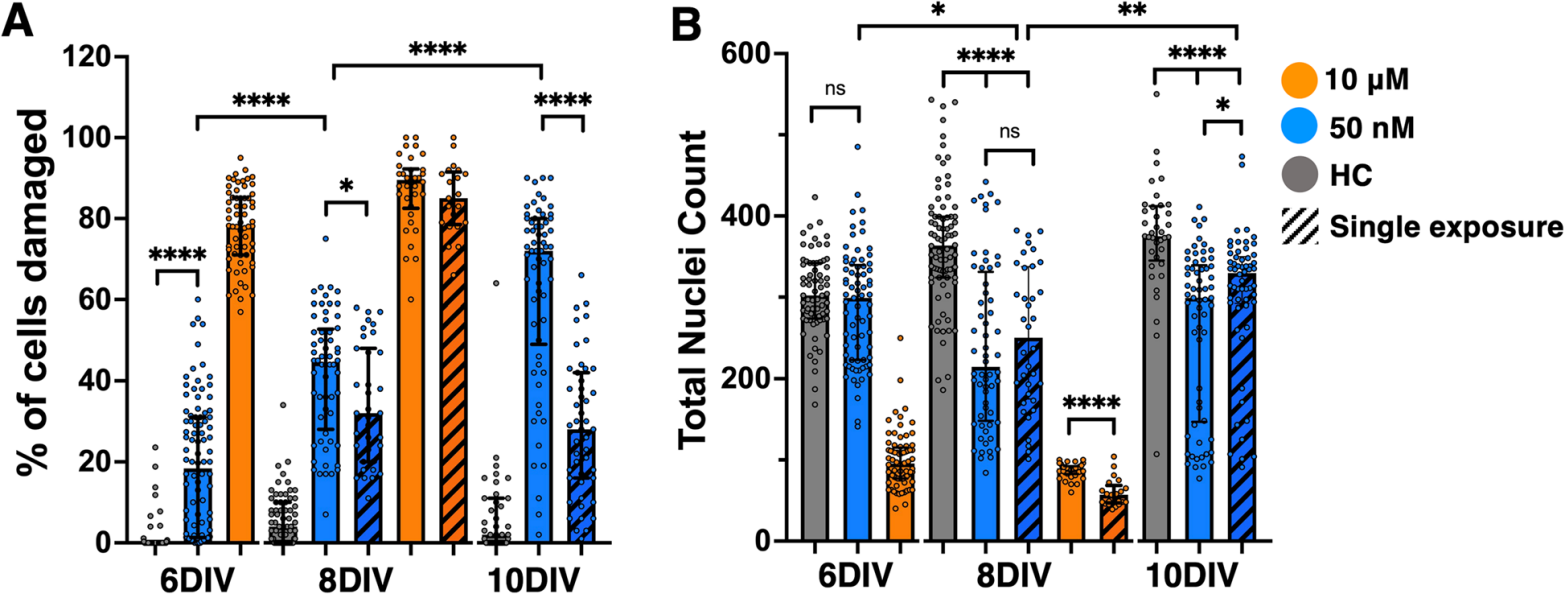
Aggregate data from all PI/DAPI experiments. **A** % cell damage and **B** total nuclei counts for all 40 × images taken in the cortex and midbrain of OWH slices in response to 50 nM and 10 μΜ ROT for single and repeat exposures. Nuclei in images from *N* = 3–6 slices were quantified, and each data point represents the result from an individual image. *p < 0.05, **p < 0.01, ****p < 0.0001

After confirming the effects of ROT exposure on whole-slice mitochondrial morphology and RNA expression, we used DAPI, a marker of all cell nuclei and PI, a marker of damaged cell nuclei, to quantify how cortical and midbrain cell populations responded to ROT exposure over time. We first aggregated all data for % cell damage and total nuclei counts for all experimental groups and observed responses that mirrored LDH results (Fig. 4A-B). PI/DAPI analysis showed consistent injury through 10DIV resulting from 50 nM exposure. The difference in injury between single and repeat exposure also became more pronounced after 8DIV (*p* < 0.0001). For all DAPI counts, healthy OWH slices retained higher populations than all ROT groups with the exception of 6DIV. The DAPI profile also revealed an inflection response at 8DIV for 50 nM-S where nuclei increased significantly (*p* < 0.01), an effect which was not seen for 50 nM-R and there was less injury over time for a single exposure. A significant difference in total nuclei between 50 nM-R and 50 nM-S was not observed until 10DIV (*p* < 0.05).

**Fig. 5:**
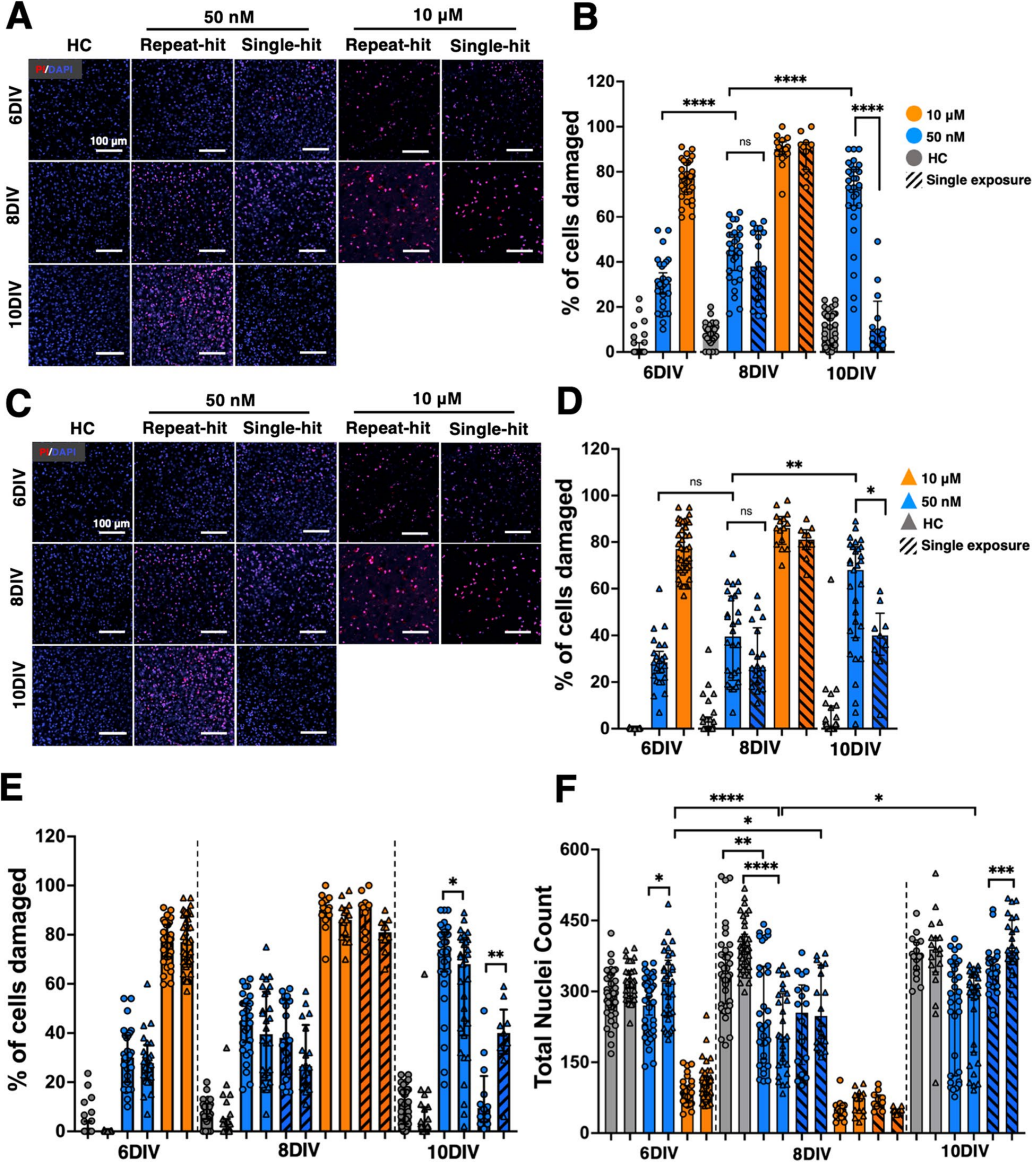
Representations of total cell density and percent of damaged cells for all assessment points. PI/DAPI imaging for representative changes in total cell density and distribution of damaged cells in the (**A**) cortex and (**C**) midbrain (*N* = 3–6 slices per condition, *n* = 5–10 images per region per OWH slices taken at 40 × magnification). Quantification of percent cell damage in the (**B**) cortex and (**D**) midbrain. Summative regional quantification of total % cell damage (**E**) and number of nuclei counts (**F**) for all groups and n the cortex (circles) and midbrain (triangles) counted manually in ImageJ following an Otsu threshold and 20-pixel size cutoff. PI-positive nuclei localized to Otsu-thresholded DAPI nuclei were manually counted with a background fluorescence cutoff kept consistent across all experimental groups. *p < 0.05, **p < 0.01, ***p < 0.001, ****p < 0.0001

To explore the regional responses to number of exposures and expand on the analysis from whole-slice LDH assessments and aggregate PI/DAPI data, we split the data by region. Representative images in each region captured the temporal dependence of cell density and % cell damage in response to ROT (Fig. 5A, C). Quantification of % cell damage and total cell counts revealed more subtle regional differences. The degree of injury experienced by cortical cells was consistently higher for a repeat exposure. Cortical cells appeared to recover more strongly by 10DIV (9.5% damaged cells) compared to midbrain cells following a single exposure (40% damaged cells) (Fig. 5B). On the other hand, in response to 50 nM-R, midbrain cells experience increasing cell damage through 10DIV (68%), comparable to % cell damage in the cortex (74%), but significantly lower (*p* < 0.05) (Fig. 5D). Despite the sustained increase in injury, midbrain cell populations generally outnumbered cortical populations (Fig. 5E). 10 µm ROT reduced overall cell density significantly (*p* < 0.0001) with very little slice available for analysis by 10 DIV (Fig. 5F). By 8 DIV, both repeat and single exposure ROT drove a significant decrease in overall nuclei counts compared to HC.

### Mature neurons and microglia respond differentially and in a spatiotemporal manner in response to ROT exposure

**Fig. 6:**
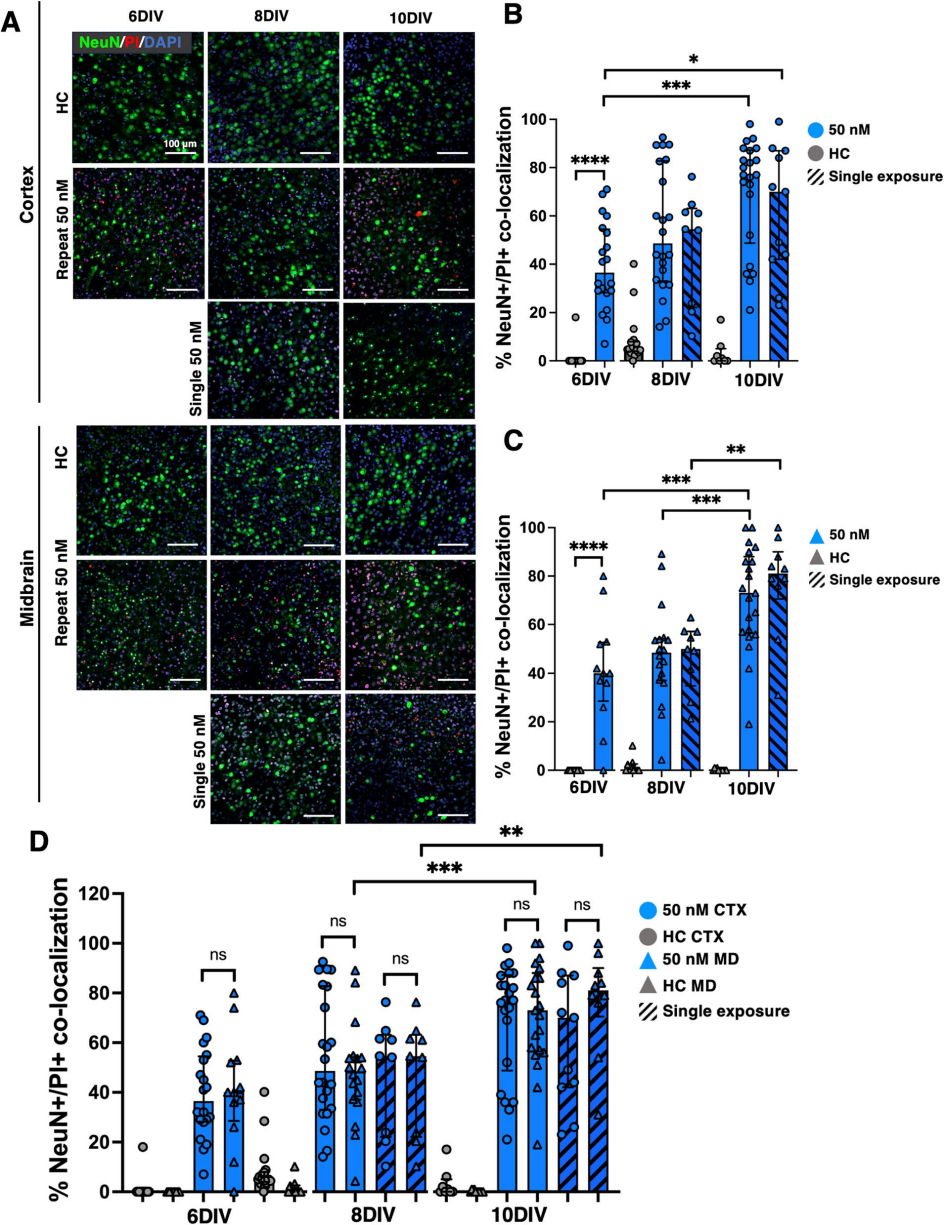
Representative images of NeuN + neurons in the (**A**) cortex and midbrain of OWH slices (*N* = 2–3 OWH slices used for each treatment group for each cell type, and *n* = 8–12 images taken at 40 × magnification per condition in each region). All OWH slices were previously co-stained with PI and and stained with DAPI after the NeuN staining regimen. Quantification of NeuN + neuron density in the cortex (**B**) and midbrain (**C**) of OWH slices. (**D**) Quantification of PI + /NeuN + co-localization in the cortex and midbrain of OWH slices. *p < 0.05, **p < 0.01, ***p < 0.001, ****p < 0.0001

**Fig. 7:**
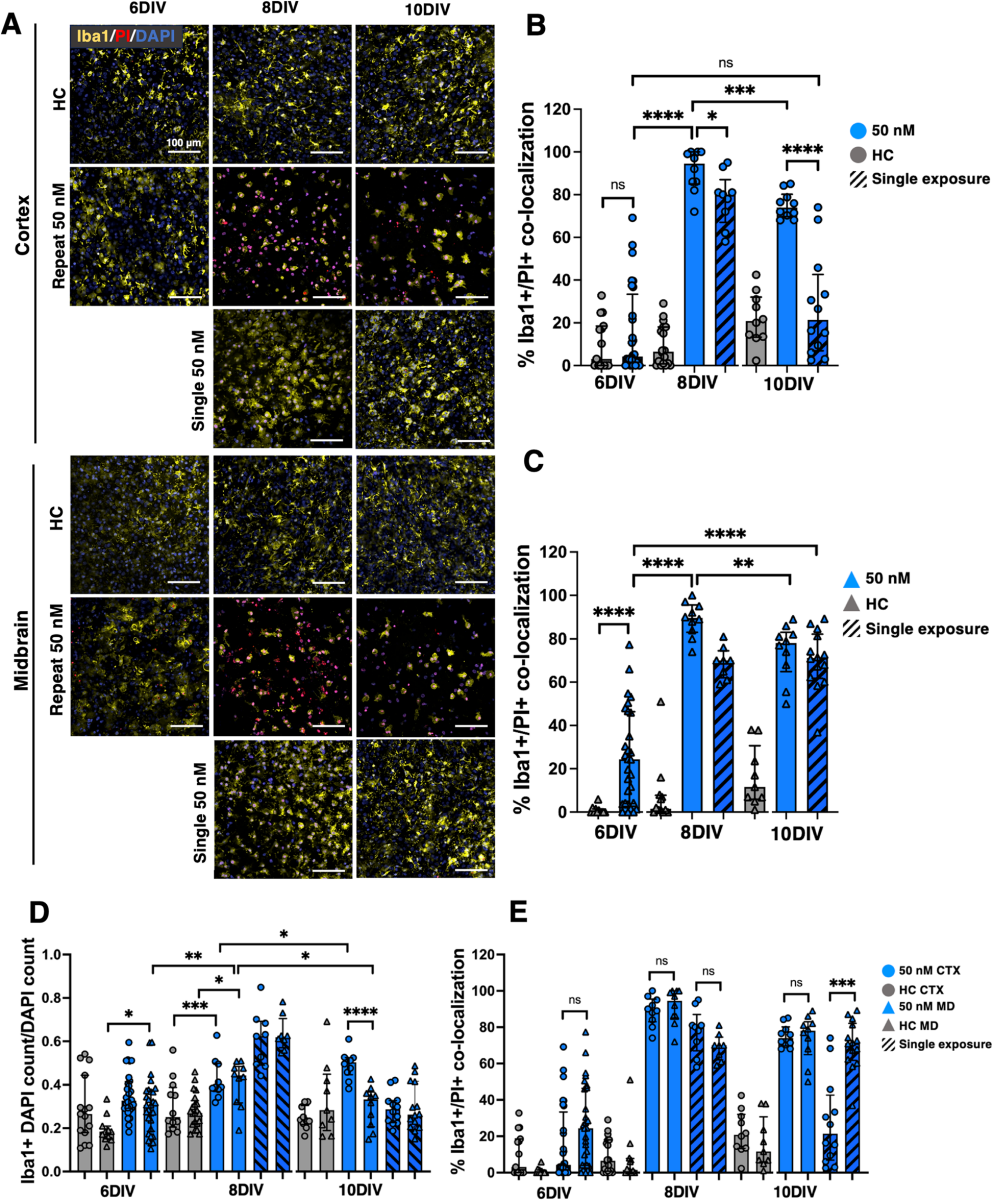
**A** Representative images of Iba1 + microglia in the cortex and midbrain of OWH slices (*N* = 2–3 OWH slices used for each treatment group for each cell type, and *n* = 8–12 images taken at 40 × magnification per condition in each region). All OWH slices were previously co-stained with PI and stained with DAPI after the Iba1 staining regimen. Quantification of Iba1 + microglia density in the (**B**) cortex and (**C**) midbrain of OWH slices. **D** Quantification of Iba1 density (DAPI + /Iba1 + co-localization) in the cortex and midbrain of OWH slices. **E** Quantification of PI + /Iba1 + co-localization in the cortex and midbrain of OWH slices. *p < 0.05, **p < 0.01, ***p < 0.001, ****p < 0.0001

To gain deeper insight into the cellular responses that may be driving these outcomes, we co-stained OWH slices with somatic markers for mature neurons (NeuN) (Fig. 6) and microglia (Iba1) (Fig. 7). Representative NeuN images are shown in Fig. 6A. Region-dependent neuronal damage was observed as a function of culture time (Fig. 6B-C). In both regions, NeuN + /PI + co-localization increased consistently between 6 and 10DIV (*p* < 0.001 for 50 nM-R in both regions) in accordance with PI/DAPI results. Additionally, in alignment with apparent recovery response observed in cortical cells following 50 nM-S, the increase in % damage between 6 and 10DIV for 50 nM-S was less significant (*p* < 0.05). The increase in NeuN + damage through 8DIV was not significant (*p* = 0.3585 in CTX, *p* = 0.4116 in MD), nor dependent on the number of exposures, but we observed regional differences from 8 to 10DIV for both exposure regimes (Fig. 6D). While PI + co-localization with NeuN in the cortex was highest at 10DIV for both exposure regimes (77% 50 nM-R, 70% 50 nM-S), the increase from 8DIV was not significant (*p* = 0.092 repeat exposure, *p* = 0.230 single exposure). Only in the midbrain did both 50 nM-R and 50 nM-S drive significant increases in NeuN damage, with slightly steeper increase in response to 50 nM-R (*p* < 0.01 single exposure, *p* < 0.001 repeat exposure). For both exposure regimes, 8DIV is a critical time point during the culturing window beyond which we saw significantly higher PI + /NeuN co-localization in both regions, as well as the effect of number of exposures in the midbrain. A 10 µM exposure resulted in significant NeuN + cell death across both brain regions (Fig. S4).

Through 10DIV, microglia in healthy OWH slices qualitatively displayed heterogeneous morphology with visible branching, reflective of a less inflammatory state, and both regions saw a pronounced shift in morphology to include a distribution of amoeboid cells following ROT exposure (Fig. 7A), reflective of a more pro-inflammatory state [62]. The presence of pro-inflammatory phenotypes was dependent on DIV and the number of exposures. At 8DIV, microglia in both regions of OWH slices given 50 nM-R adopted primarily amoeboid distributions, with median circularities 0.599 and 0.567 in the cortex and midbrain, respectively. Microglia OWH slices given 50 nM-S sustained median circularities closer to healthy controls (0.356 CTX, 0.325 MD), and qualitatively showed a more mixed-morphology population similar to healthy controls. By 10DIV, microglia in both regions of slices given a single exposure appeared to shift towards heterogeneous morphology populations more consistent with healthy controls. Quantitative data on circularity and aspect ratio of microglial populations confirmed that significant morphological differences separated the midbrain of single-exposure and healthy OWH slices by 10DIV (*p* < 0.01) (Fig. S6).

Microglial co-localization with PI + nuclei increased significantly by 6DIV in the midbrain (*p* < 0.0001), whereas the cortex was more resistant over the first 48 h of response. By 8DIV, however, both regions saw a comparable increase in Iba1 + /PI + co-localization independent of the number of exposures (Fig. 7B-C). Both regions saw significant decline in Iba1 + /PI + co-localization from 8 to 10DIV, although the decrease in the midbrain was less significant (*p* < 0.01) compared to that observed in the cortex (*p* < 0.001). Within the 50 nM-S group, the regional differences in PI + co-localization over the culturing window were most distinct by 10DIV. Microglial co-localization with PI + nuclei in the cortex recovered through 10DIV (21.4%), while microglia the midbrain sustained more PI + co-localization (71.2%). Additionally, PI + co-localization in the cortex between ROT-exposed microglia at 6DIV and 50 nM-S microglia at 10DIV was close in value but not significant (*p* = 0.0567). The opposite was observed in the midbrain, where the midbrain showed significantly higher PI + co-localization by 10DIV following a single exposure (*p* < 0.001) compared to levels at 6DIV (Fig. 7E). Generally, microglia in the midbrain retained higher PI + co-localization regardless of the number of exposures. A single exposure demonstrated that cortical microglia initially show stronger resistance and ultimately recovered close to healthy levels, but still experience a peak in PI + co-localization at 8DIV regardless of the number of exposures. Similar to the NeuN cellular response, 10 µM ROT exposure resulted in high microglial death (Fig. S5).

Both regions of ROT-exposed OWH slices displayed elevated microglial densities relative to healthy OWH slices by 8DIV, with 50 nM-S microglial density (0.624 CTX, 0.619 MD) outnumbering those in 50 nM-R OWH slices (0.389 CTX, 0.443 MD). Cortical microglial density increased only slightly from 8 to 10DIV following repeat exposure (+ 0.115, *p* < 0.05) and decreased significantly by 10DIV following a single exposure (-0.337, *p* < 0.0001), but remained higher than microglial density in the midbrain following repeated exposure (0.332). A single exposure resulted in a reduction in microglial density by 10DIV, but the reduction was only significant in the midbrain (*p* < 0.01). However, despite the lesser number of microglia, PI + co-localization remained significantly higher in the midbrain by 10DIV. Contextualizing the responses of both neurosn and microglia highlights region-dependent susceptibility and inflammatory changes depending on time in vitro and the number of ROT exposures. Summative tables of median (± IQR) % PI + co-localization of neurons and microglia, as well as microglial density are provided for healthy slices (Table S1), single 50 nM ROT exposure (Table S2), repeat 50 nM ROT exposure (Table S3), and single and repeat 10 µM exposure (Table S4). Summative analysis of Iba + , NeuN + , and PI + quantification is visualized in Fig. S7.

### Extracellular microstructure dynamically and regionally changes as a result of ROT exposure

**Fig. 8:**
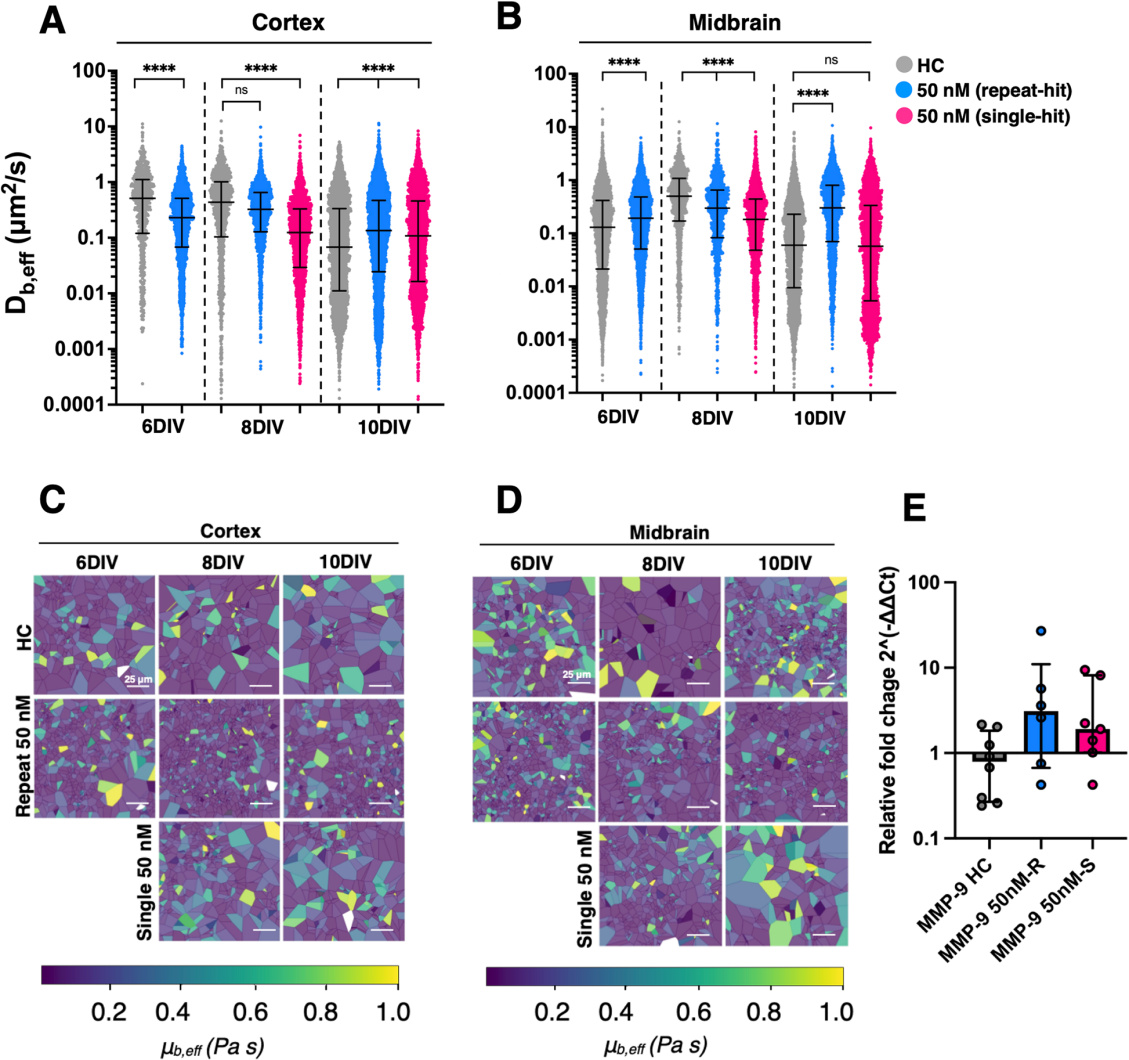
Distributions of effective diffusion coefficients (*D*_*b,eff*_) comparing particle diffusion in healthy and 50-nM exposed OWH slices through 10DIV in the (**A**) cortex and (**B**) midbrain. Representative mosaic heatmaps of Stokes–Einstein viscosities (Pa s) in the (**C**) cortex and (**D**) midbrain experienced by 40 nm PS-PEG particles at 37 °C. Particles were tracked in 2–3 slices per each condition, with 3–5 videos were collected in each region per condition, resulting in > 2,000 trajectories per condition. All videos were acquired at 67 frames per second, 100 × magnification, and within 1 h following particle incubation. Representative effective diffusion coefficients and viscosities are calculated at a trajectory lag time of τ = 0.1 s. Mean-squared displacement profiles from 0 < τ = 1.0 s for all groups are provided in supplemental Fig. 6. (**E**) Expression of MMP-9 at 8DIV for 50 nM-R and 50 nM-S, as quantified by RT-qPCR, where each data point corresponds to group of three OWH slices pooled together for RNA extraction. ****p < 0.0001

To characterize steric and micro-viscosity changes in the extracellular microenvironment in response to ROT exposure compared to normal OWH slice culturing, we performed MPT with 40 nm PS-PEG nanoparticle probes at each time point throughout ROT exposure. Nanoparticle probe physicochemical properties are reported in Table S5. Distributions of *D*_*b,eff*_ were split by region and plotted over time in response to 50 nM-R and 50 nM-S (Fig. 8A-B). In the cortex, the trend in particle diffusion through 10DIV mirrored that which was seen in previous studies in healthy P10 OWH studies [44]. Following 48 h of 50 nM ROT exposure, particles in the cortex moved significantly slower (*D*_*b,eff*_ = 0.230 μm^2^/s) compared to those in healthy OWH slices at 6DIV. Quantifying local viscosity in the cortex at 6DIV revealed a more viscous microenvironment following ROT exposure, which increased following 50 nM-R at 8DIV (*D*_*b,eff*_ = 0.324 μm^2^/s). Particles diffused even slower in OWH slices following 50 nM-S, although particles in the cortex of 50 nM-S OWH slices moved faster at 8DIV compared to their diffusion at 6DIV. By 10DIV, particles in the cortex of 50 nM-R OWH slices showed faster diffusion (*D*_*b,eff*_ = 0.135 μm^2^/s) compared to particles in healthy OWH slices.

The effect of ROT on particle diffusion in the midbrain was initially opposite that which was observed in the cortex but mirrored the cortex through 10DIV. By 8DIV, particle behavior in the midbrain resembled the trends observed in the cortex, with slower diffusion for 50 nM-R and 50 nM-S, respectively. Additionally, at 10DIV, not only did the trends in particle diffusion in the midbrain mirror those in the cortex, but the differences relative to diffusion in healthy OWH slices were more pronounced (Fig. S8). Micro-rheology of the local extracellular diffusion environments in each condition, represented as a brain effective viscosity *μ*_*b,eff*_ (Pa s), was calculated (Table 2) and visualized using representative mosasic heatmaps. Viscosity calculations revealed a similar trend between both regions for a single-hit exposure, where the increase in viscosity at 6DIV remained relatively constant through 8DIV, but increased sharply at 10DIV. Repeat exposure also resulted in microenvironment viscosity values more consistent with those calculated in healthy tissue at 10DIV.

**Table 2:** Median effective brain Stokes–Einstein viscosities (μ_b,eff_ × 10^2^_,_ Pa s) calculated at a trajectory lag time of τ = 0.1 s with average hydrodynamic diameter 54.8 nm and T = 37 °C

	**Cortex**			**Midbrain**		
DIV	6DIV	8DIV	10DIV	6DIV	8DIV	10DIV
HC	1.75	2.06	13.2	6.90	1.79	14.9
Single-hit	3.90	7.23	8.23	4.66	3.02	2.97
Repeat-hit	DNC	2.76	6.64	DNC	4.90	15.7

We screened expression of matrix metalloproteinase-9 (MMP-9) at 8DIV in HC, 50 nM-R and 50 nM-S OWH slices to probe the relationship between ROT-driven remodeling of the extracellular matrix (ECM) and particle behavior. We anticipated that that higher levels of the protease would lead to ECM breakdown and lessen steric resistance to diffusion, expected that MMP-9 expression would correlate with particle diffusivity, especially since the particle diffusion pattern in both regions was similar. However, ROT-exposed slices at 8DIV saw higher expressions of MMP-9 in both exposure groups relative to healthy OWH slices despite slower particle diffusion. We observed an increase in MMP-9 expression from 50 nM-R relative to HC OWH slices (*p* = 0.059) and a slightly lesser increase from 50 nM-S relative to HC OWH slices (*p* = 0.072).

## Discussion

**Fig. 9:**
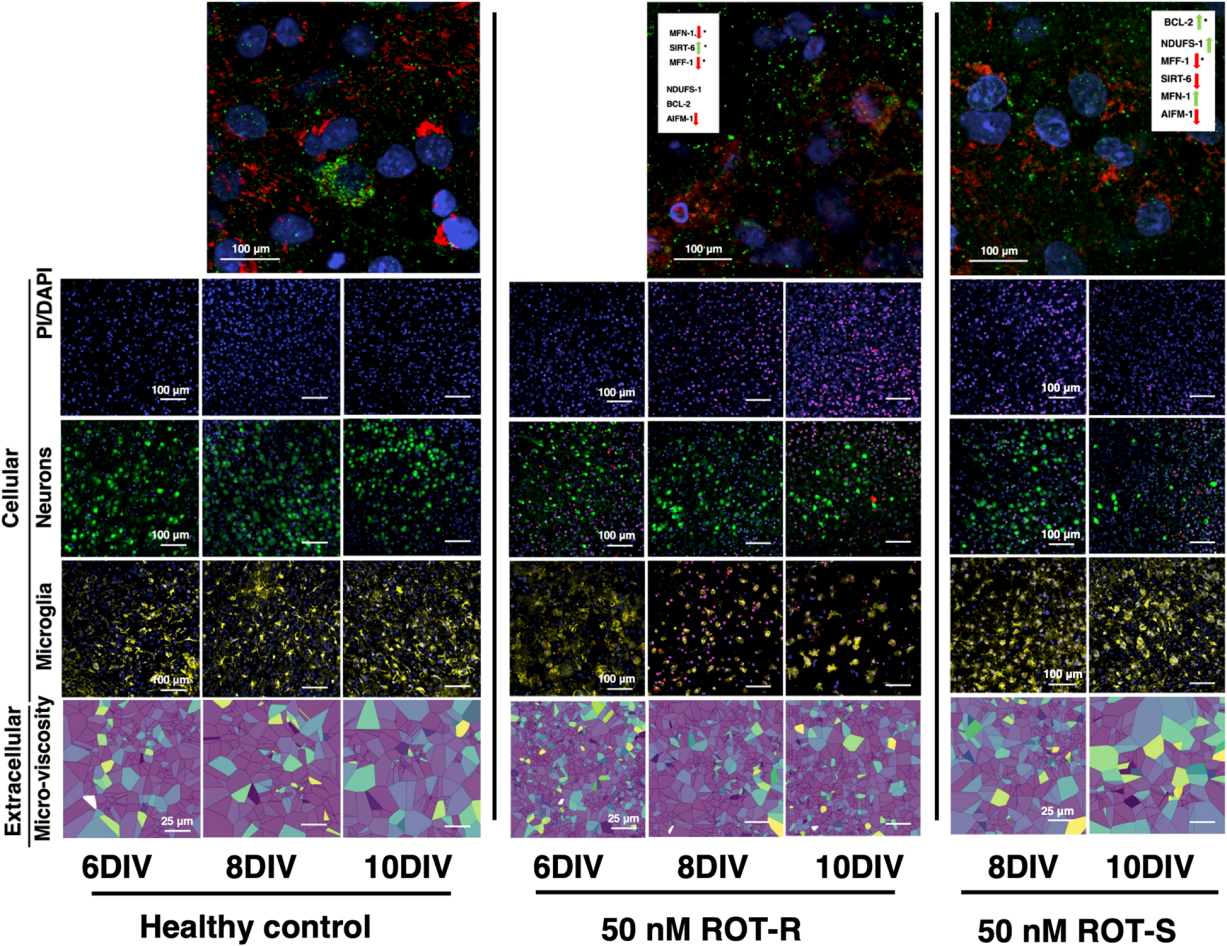
Summary of multi-scalar results as a function of culture time and number of 50 nM ROT exposures. Changes in gene expression at 8DIV relative to healthy OWH slices for each experimental group reported in MitoTracker/Complex I images (green arrows = increase, red arrows = decrease, asterisks = significance level). Representative images of aggregate cortex and midbrain data for each measurement are provided

ROT exposure is commonly used in models of the central nervous system to study the relationship between mitochondrial abnormalities and neurodegenerative pathologies. Emerging clinical evidence also points to mitochondrial integrity as one mediator of the developing brain’s response to stress and injury, and therefore we extended a ROT model framework to neonatal OWH brain slices. To our knowledge, ROT exposure has not been previously investigated in organotypic brain tissue cultures ontaining multiple brain regions at term equivalency. This study establishes the utility of ROT in OWH slice cultures as a compliment to in vitro and in vivo ROT models, and also demonstrates the exposure regime and time dependence of multi-scalar responses to the mitochondrial poison (Fig. 9).

Our results showed an injury dependence on both ROT exposure level and culture time. We initially did not expect to see any significant injury from 1 nM ROT because the IC_50_ for ROT-complex I binding in rat brain tissue is reported to range between 8.2 – 19.9 nM, depending on region [5]. We also estimated an intracellular concentration of at least 10 nM to inhibit complex I based on estimates of complex I levels in astrocytes (2.2 × 10^5^ molecules/cell, 6.6 × 10^4^ μm^3^/cell volume) and assuming a 1:1 binding stoichiometry [63, 64]. However, at 10DIV, the injury from 1 nM exposure was significant, suggesting a time dependence. The accumulation of injury resulting from repeated exposure to sub-IC_50_ ROT has also been shown in other model systems [54]. Our LDH results for the 1 nM group parallel these findings. The 50 nM and 100 nM groups showed similar injury levels at 6DIV, which aligns with the similar levels of acute injury (24 h) observed in SH-SY5Y neuron cultures for these exposure levels [59]. The difference in injury between the 50 nM and 100 nM groups diverges significantly between 6 and 10DIV, which highlighted the compounding effects of exposure level and exposure time on ROT injury in OWH slices. The time-dependent injury, despite sub-threshold exposure, may be attributed to ROT accumulation in the lipophilic environment of the mitochondrial inner membrane. While the level of injury from 50 nM exposure remained consistent, the relative change in injury from 100 nM exposure increased through 10DIV and approached 10-μΜ injury levels. Imaging data showing varied distribution of mitochondrial health across injured groups also suggested that our exposure regime was sufficient to drive changes in mitochondria without fully knocking out function. This distinction informed our choice to use 50 nM ROT to explore regional and multi-scale responses in a regime of consistent low-grade injury and utilize 10 μΜ as a positive control for ROT-driven cell death.

Prior to this study, most in vitro ROT organotypic models focus on individual subcortical structures. We anticipated regional differences due to evidence of region-dependent metabolic activity and region-specific outcomes from mitochondrial impairment [16–18, 20, 23], which can affect the developing brain through a variety of avenues [21, 24–28, 65, 66]. Complex I levels in rat brain tissue are also shown to be region- and cell-type dependent [67, 68]and ROT-complex I IC_50_ concentrations vary regionally, with the highest reported in midbrain structures (19.9 nM) [5]. Relative regional susceptibilities have not been extensively explored in culture, but midbrain and striatal tissue homogenates experienced greater loss in glutathione peroxidase activity following ROT incubation compared to frontal cortex and hippocampal homogenates [69]. Therefore, while we expected and confirmed multi-region cytotoxicity profiles would mirror the trends in whole-slice cytotoxicity data, we also anticipated regional differences. Time in culture defined by DIV accelerated the regional differences more than any other factor in the change in cell death following single or repeat 50 nM exposure. Through the lens of total cell numbers, culture time also accelerated regional differences and showed an apparent inflection response at 8DIV.

We hypothesized that the tradeoffs been cell numbers and % cell damage may be attributed to the microglial response to neuronal damage, an effect which has previously been shown in vitro [57]. The comparable neuronal cytotoxicity levels between single-hit and repeat-hit ROT over the culturing window might be attributed more to microglial interactions, since microglia are thought to be more resistant to complex I driven injury. ROT promotes reactive oxygen species (ROS) generation directly in neurons and via microglial activation, so we expected initial neuronal cytotoxicity followed by a microglial response and increased neuronal damage over time. We saw this within the first 48 h of 50 nM ROT exposure with comparable neuronal damage between the two regions alongside an increase in microglial co-localization with PI + nuclei. Despite comparable neuronal injury, the midbrain showed a higher density of microglia, a response that peaked at 8DIV. Interestingly, this time point is also where the inflection responses in neuron cytotoxicity occurred, after which midbrain neurons experienced a steeper increase in injury compared to cortical neurons independent of the number of exposures. The degree of injury between the two regions was not significant, but achieving significant damage within 6 days following 50 nM ROT exposure aligns with a study using organotypic substantia nigra cultures where a single 50 nM exposure comparably shortened dopaminergic neuron processes and changed morphologies after 1 week [54]. While these results indicate that midbrain cells may be more susceptible to ROT exposure at longer culturing times, the interactions driving this susceptibility remain speculative. Microglial densities decreased by 10DIV—more so for a single exposure compared to a repeat exposure—and the remaining microglia in the midbrain retained significantly higher PI + co-localization, suggesting a more lasting effect of the inflammatory response. Futher work performing analysis on microglia co-localized with PI using spatial transcriptomics or single cell sequencing techniques could further parse out cell–cell interactions contributing to time-dependent regional differences in cell damage.

Alterations to the extracellular microenvironment are also seen in models of injury and disease states that involve mitochondrial abnormalities. Changes to the extracellular microenvironment can occur through structural remodeling of the ECM, such as rearrangement, degradation, or deposition, protein expression, fluctuations in local ionic strength and pH, changes in extracellular space volume due to cell swelling, death, proliferation, morphological changes, all of which happen dynamically in healthy and injurious states [70]. Mitochondria play specific roles in activating pathways that result in altered ECS volume and ECM properties, which can affect the behavior of extracellularly diffusing probes. Mitochondrial dysfunction can drive dysregulation of intracellular ion concentrations, which can result in neuronal and glial cell swelling and effectively decrease ECS volume [67]. By 8DIV, there was a morphological shift to a distribution of pro-inflammatory cells in ROT-exposed slices, and an increase in microglial density. This inflammatory shift likely contributes to ECM remodeling through the release of pro-inflammatory cytokines and enzymes like metalloproteinases, further impacting the extracellular microenvironment. Complex I inhibition in vivo is also shown to result in upregulation of ECM-digesting enzymes*,* such as MMPs 3 and 9 [68]. MMPs degrade proteolytic backbones of the brain ECM and can alter the viscous environment experienced by extracellulary diffusing probes, an effect which has been shown in tissue from an in vivo synucleinopathy model [71]. Higher MMP activity in response to ROT could result in an increase in viscosity from increased concentrations of digested or fractionated ECM proteins, an effect we did measure in our MPT studies which could slow diffusion. MMP-9 is well-studied in the brain and has enzymatic affinity towards constituents of the extracellular matrix as well as roles in neural cell adhesion and organization [72, 73]. We suspect the latter may help explain why our MMP-9 expression results contradicted our hypothesis, as cytoarchitecture remodeling was likely needed to compensate the microglial responses at this time point. MMP-9 activation can also represent a concerted effort of other MMPs [74] and we chose MMP9 as an initial catch-all probe for the presence of microenvironment-remodeling molecules as it pertains to altered nanoparticle diffusion.

We can assess the change in diffusivity through the lens of cell density, which we expect to show proportionality with effective diffusivity. The decrease in cell density from 6DIV through 8DIV aligns with increased particle diffusivity in both regions, and the recovery of cell density through 10DIV following a single exposure aligns with decreased particle diffusivity over time. Diffusivity trends for repeat exposure are less explained by changes in cell density, where diffusivity continued to increase through 10DIV in the midbrain (but not the cortex) despite nonsignificant changes in cell density. This discrepancy may be explained by the microglial response from 8DIV through 10DIV. Higher PI + co-localization and density of microglia in the midbrain could explain the regional variability in particle diffusion observed in our study. Microglial density in the midbrain was higher than the cortex, compared to comparable density at 8DIV. Additionally, higher density of microglia with pro-inflammatory morphologies may effectively decrease extracellular volume fraction and correlate with lower particle diffusivity. Disruption to cellular energy metabolism has extracellular consequences, and changes in ECM stiffness can also alter mitochondrial morphology and integrity [75]. Our MPT results in OWH slices supported these findings, capturing differences in tissue microstructure resulting from ROT exposure and mapping these data with cellular and mitochondrial behavior. Changes in ECM stiffness can also alter mitochondrial morphology and integrity [76]. Downregulation of MFF-1 for ROT groups relative to healthy tissue defied our expectations, but we also have to recognize that the timescales of ECM remodeling and RNA expression are likely very different, and standalone expression may not be enough to describe the full state of the system. For example, the upregulation of MFN-1 at the same time point which might suggest a compensatory response, despite a stiffer microenvironment. While there are multiple avenues by which diffusing nanoparticles experience a different microenvironment, these data are limited to descriptive and correlative analysis. Future work could probe causational relationships that contribute to diffusive differences by quantifying hyaluronan fractionation and ECM protein expression, nanoparticle uptake and clearance, osmotic pressure and ionic strength, and cell-specific proliferation [44, 71, 75, 77–79].

This study established a complimentary model system to common in vitro and in vivo ROT models to study mitochondrial dysfunction in the neonatal brain with high experimental throughput. We demonstrate the ability of OWH slice cultures exposed to ROT to capture time-, region-, and severity-dependent responses across relevant scales. However, it is important to acknowledge some key limitations of the model and its interpretations. It remains a challenge to synthesize and interpret data collected across a range of length and time scales. The high-throughput nature of the organotypic platform naturally lends itself to focused mechanistic studies to help resolve the length and time scales over which slices recapitulate these functional states. In terms of model design, the scope of these studies does not capture other aspects of mitochondrial responses in the neonatal brain, primarily the influences of sex and developmental age where we might expect different outcomes. Mitochondrial electron transport chain (ETC) protein expression, respiratory capacity, and susceptibility to oxidative stress is shown to vary with sex, developmental age, and region [23, 80]. These characteristics are also shown to be injury dependent. In another study comparing sex differences in neonatal hypoxic-ischemic injury, P8 female rats showed stronger recovery in mitochondrial ETC protein expression following treatment [19]. Other studies generally highlight higher resistance to oxidative stress and less ROS leakage in female rodents, with some hypothesizing higher differentiation in mitochondria as a reason for higher resilience [22]. The decision to use healthy female rats at P10-P11 comes from previously published work [44]. We would expect sex-based differences in response to ROT based on reported sex-based differences in mitochondrial differentiation and electron transport chain protein expression. Therefore, to eliminate the influence of sex-based differences, we only used female tissue donors. Future work can incorporate male rats and different postnatal ages into the study design to consider sex-based and age-based differences if it is desired to implement ROT exposure to study therapeutic efficacy of drugs that target mitochondrial regulation in neonates or pediatric populations. While the postnatal age used in this study is well-suited for longer culturing times, we also recognize the limitations in focusing on a single developmental age. Developmental age-based differences should be considered, but for OWH slices specifically, the tradeoff between donor age and long-term culturability must also be acknowledged [44].

Most ROT models study the effects on neurons and how microglial interactions modulate neuronal survival, therefore we chose to focus our studies to these cell types. Microglia are shown to depend more on energy support from mitochondrial oxidative phosphorylation (OXPHOS) compared to other glial cell types [2, 81]. However, it is also likely that other neural cells interact with neurons and microglia in ways that contribute to the regional and exposure-dependent differences seen [2, 29, 64, 82]. Future work should consider other cell types, such as astrocytes and oligodendrocytes, when studying neonatal mitochondrial abnormalities or developing cell-targeted treatments. Astrocytes are thought to depend less on OXPHOS and more on glycolytic machinery, rendering them less susceptible to a complex I-based insult [2]. Astrocytes are also known to participate in intercellular transfer of mitochondria, which may be neuroprotective or trigger cell death depending on directionality and the cell types involved [83]. Oligodendrocytes utilize both forms of energetic machinery, but rely more on mitochondrial OXPHOS for the intense energy cost of myelination (3.3 × 10^23^ ATP/1 g myelin), which position them as an important cell type to study in a regime of neurodevelopmental mitochondrial dysfunction [84]. However, studying oligodendrocyte response to ROT may be more robust in an OWH slice model with higher white-to-gray matter ratios, such as the ferret or piglet brain. Improved understanding of the roles of multiple cell types in mitochondrial response following complex I inhibition could reveal other avenues for therapeutic intervention and evaluation of mitochondrial performance in developmental brain injury.

## Conclusions

We adapted the complex I inhibitor ROT for driving mitochondrial dysfunction in neonatal brain slices from term-equivalent rodents, focusing on establishing a robust experimental framework using the OWH culture platform. We identified 50 nM ROT as effective dose to induce consistent injury without compromising overall slice health. By 8DIV, we observed distinct responses following a repeat and single exposure of 50 nM ROT that had compounded effects over culture time. Notably, both exposures induced mitochondrial morphological changes and spatial disorientation at 8DIV. Expression at 8DIV of select genes related to mitochondrial regulation, morphology, and apoptosis pathways suggested an effect of exposure level on ability to activate apoptotic pathways and restore morphology. At the cellular level, cortical cells in response to ROT showed sustained increases in cytotoxicity, contrasting with an initially subdued injury profile in the midbrain. Quantifying neuronal damage, microglial localization with damaged cells, and microglial density suggested differences in microglial responses and microglia-neuron interactions that contribute to regional differences in cell damage and density in response to exposure regime and culture time. Behavior of extracellularly diffusing nanoparticle probes also revealed regional, time-, and exposure-dependent differences that can be explained in part by changes in microglial density, morphology, and pro-inflammatory consequences. These findings underscore the complex interplay of region-specific vulnerabilities and time in response to mitochondrial dysfunction. Future research can explore sex and OWH donor age-related variables, other cell types, and mitochondrial oxygen consumption and glycolytic function to enhance our understanding of mitochondrial responses in the presence of pathology in the developing brain.

## Supplementary Information

Supplementary Material 1.

## Data Availability

All datasets supporting the conclusions of this article are available upon request. Please contact the corresponding author for access. Datasets for MPT, as well as all code, are available on github, with links included in the methods.

## Abbreviations

OWH: Organotypic whole-hemisphere
ROT: Rotenone
SCM: Slice culture media
RSCM: Slice culture media with rotenone
DMSO: Dimethyl sulfoxide
RDMSO: Dimethyl sulfoxide with rotenone
DIV: Days in vitro
P: Postnatal age (days)
LDH: Lactate dehydrogenase
RT-qPCR: Reverse transcription quantitative polymerase chain reaction
PI: Propidium iodide
DAPI: 4',6-Diamidino-2-phenylindole
CTX: Cortex
MD: Midbrain
NeuN: Hexaribonucleotide binding protein-3, mature neuron marker
Iba1: Ionized calcium binding adaptor molecule 1, microglia marker
MPT: Multiple-particle tracking
ECM: Extracellular matrix
ECS: Extracellular space
D_b__,eff_: Brain effective Stokes–Einstein diffusion coefficient
μ_b__,eff_: Brain effective Stokes–Einstein viscosity
ETC: Electron transport chain
OXPHOS: Oxidative phosphorylation
ATP: Adenosine triphosphate
NADH/NAD +: Nicotinamide adenine dinucleotide
MMP-9: Matrix metalloproteinase-9
NS: Not significant
