# Supplementary material for: A rotenone organotypic whole hemisphere slice model of mitochondrial abnormalities in the neonatal brain
Source: J Biol Eng. 2024 Nov 14;18:67. doi: 10.1186/s13036-024-00465-w (PMC11566268; doi:10.1186/s13036-024-00465-w)
Supplement: Supplementary file 1 — Supplementary Material 1. [file 13036_2024_465_MOESM1_ESM.pdf]

Table S1. Summary of median (±IQR) values for total cell count, % cell damage, % microglia-PI co-localization, % microglia-DAPI co-localization, and % neuron-PI co-localization in healthy OWH slices. Number of asterisks (*) following the IQR denotes extent of non-Gaussian distribution if the dataset failed the D'Agostino-Pearson omnibus K2 test for normality. Normally distributed datasets are denoted with a hash symbol (#) following the IQR.

	6DIV HC		8DIV HC		10DIV HC	
	Cortex	Midbrain	Cortex	Midbrain	Cortex	Midbrain
Median cell count (±IQR)	290 ± 74.0#	315 ± 58.0#	334.5 ± 103.7#	391 ± 61.5#	374 ± 57.5#	389 ± 73*
Median % cell damage (±IQR)	0.00 ± 4.10****	0.00 ± 0.00****	8.00 ± 6.25#	0.00 ± 5.50****	11.0 ± 14***	2.00 ± 9.75****
Median % NeuN-PI nuclei co-localization (±IQR)	0.00 ± 0.00****	0.00 ± 0.00	4.92 ± 4.04****	0.00 ± 2.57****	0.00 ± 1.00***	0.00 ± 0.00**
Median % Iba1-PI nuclei co-localization (±IQR)	5.10 ± 20.03#	0.00 ± 1.52****	1.00 ± 3.00#	0.00 ± 1.00****	20.85 ± 18.72#	11.65 ± 24.81#
Median % Iba1/nuclei co-localization (±IQR)	26.5 ± 26.3#	18.4 ± 5.90*	27.2 ± 17.9#	31.6 ± 8.90#	23.6 ± 8.80#	28.3 ± 25.9#

Table S2. Summary of median (±IQR) values for total cell count, % cell damage, % microglia-PI co-localization, % microglia-DAPI co-localization, and % neuron-PI co-localization following a single 50 nM exposure. Number of asterisks (*) following the IQR denotes extent of non-Gaussian distribution if the dataset failed the D'Agostino-Pearson omnibus K2 test for normality. Normally distributed datasets are denoted with a hash symbol (#) following the IQR.

	8DIV 50 nM (single exposure)		10DIV 50 nM (single exposure)	
	Cortex	Midbrain	Cortex	Midbrain
Median cell count (±IQR)	255 ± 168#	248 ± 166*	342 ± 54.0#	387.5 ± 88.0#
Median % cell damage (±IQR)	38.0 ± 30.3*	26.5 ± 23.8#	9.50 ± 18.8*	40.0 ± 18.0#
Median % NeuN-PI nuclei co-localization (±IQR)	54.6 ± 41.1#	50.0 ± 22.4#	70.0 + 45.0#	81.0 ± 19.5*
Median % Iba1-PI nuclei co-localization (±IQR)	80.0 ± 20.0#	70.0 ± 12.5#	21.4 ± 35.7#	71.2 ± 20.4#
Median % Iba1/nuclei co-localization (±IQR)	62.4 ± 20.3#	61.9 ± 13.4#	28.7 ± 10.1#	26.2 ± 21.0#

Table S3. Summary of median (±IQR) values for total cell count, % cell damage, % microglia-PI co-localization, % microglia-DAPI co-localization, and % neuron-PI co-localization following repeated 50 nM exposure. Number of asterisks (*) following the IQR denotes extent of non-Gaussian distribution if the dataset failed the D'Agostino-Pearson omnibus K2 test for normality. Normally distributed datasets are denoted with a hash symbol (#) following the IQR.

	6DIV 50 nM		8DIV 50 nM (repeated exposure)		10DIV 50 nM (repeated exposure)	
	Cortex	Midbrain	Cortex	Midbrain	Cortex	Midbrain
Median cell count (±IQR)	281 ± 105#	319 ± 117.3#	228.5 ± 196.5*	205 ± 150.8*	296 ± 237**	303 ± 164*
Median % cell damage (±IQR)	30.0 ± 19.0#	27.8 ± 14.2#	45.0 ± 15.8#	39.5 ± 36.0#	74.0 ± 16.0***	68.0 ± 39.0#
Median % NeuN-PI nuclei co-localization (±IQR)	36.5 ± 26.3#	40.0 ± 24.3#	48.7 ± 49.8#	48.5 ± 17.2#	77.0 ± 38.5#	73.0 ± 31.5#
Median % Iba1-PI nuclei co-localization (±IQR)	4.24 ± 31.9*	24.3 ± 42.4#	94.5 ± 15.0#	89.5 ± 12.5#	74.0 ± 10.4#	78.0 ± 18.1#
Median % Iba1/nuclei co-localization (±IQR)	32.7 ± 12.2#	30.6 ± 18.5#	38.9 ± 13.0#	44.3 ± 16.8#	50.4 ± 7.86#	33.2 + 16.0#

Table S4. Summary of median (±IQR) values of total cell count and % cell damage (by region and aggregate) following single and repeat 10 µM exposure. Number of asterisks (*) following the IQR denotes extent of non-Gaussian distribution if the dataset failed the D'Agostino-Pearson omnibus K2 test for normality. Normally distributed datasets are denoted with a hash symbol (#) following the IQR.

	6DIV 10 µM			8DIV 10 µM (single exposure)			8DIV 10 µM (repeated exposure)		
	Cortex	Midbrain	Aggregate	Cortex	Midbrain	Aggregate	Cortex	Midbrain	Aggregate
Median cell count (±IQR)	82.5 ± 36.1#	109 ± 36.0****	95.5 ± 38.0****	51.5 ± 23.0#	74.0 ± 36.0#	57.0 ± 22.0****	65.0 ± 24.0***	48.0 ± 7.00#	89.5 ± 9.75*
Median % cell damage (±IQR)	77.0 ± 13.0#	77.0 ± 19.6*	78.2 ± 14.2#	91.0 ± 12.0#	81.0 ± 8.50#	85.0 ± 13.0*	90.0 ± 5.50***	86.0 ± 12.0#	58.5 ± 40.2#

Table S5. Physicochemical properties for the two batches of 40 nm polystyrene-(poly)ethylene glycol (PS-PEG) nanoparticles used for MPT measurements in this study.

40 nm PS-PEG	Number mean size (nm) ± SEM	PDI ± SEM	Zeta potential (mV) ± SEM
Batch 1	55.2 ± 2.83	0.08 ± 0.014	-2.56 ± 0.31
Batch 2	54.6 ± 1.92	0.06 ± 0.015	-3.13 ± 0.68

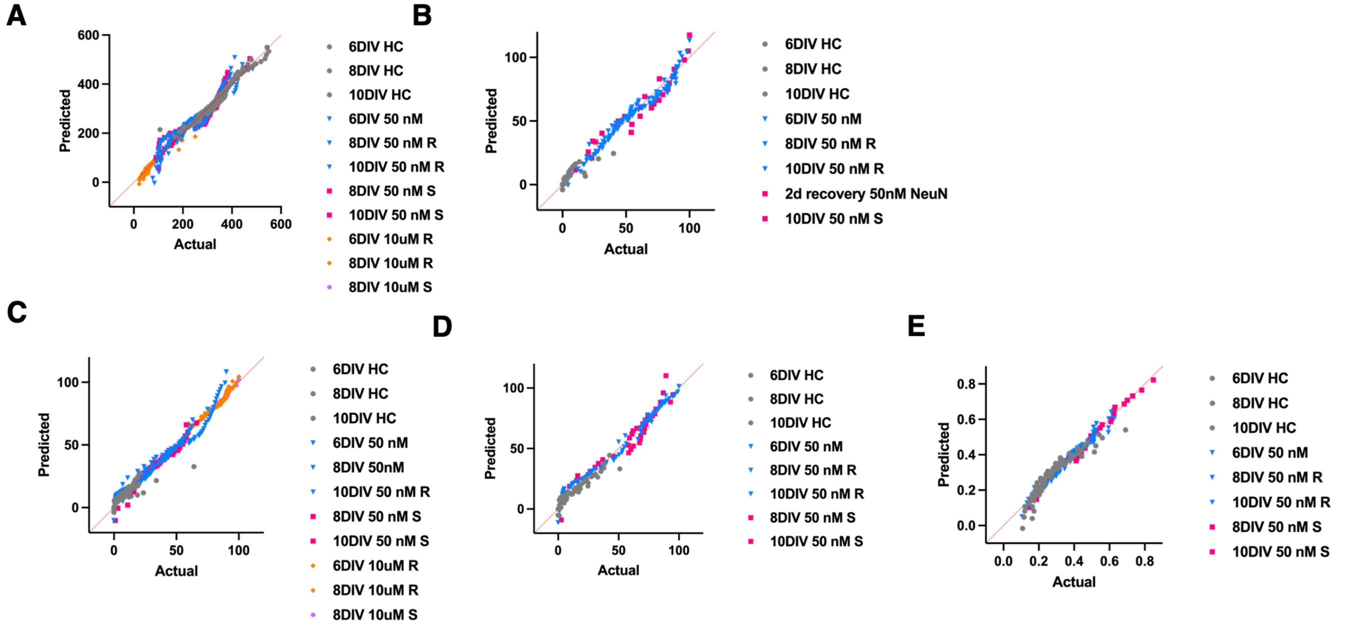

Figure S1. Normality QQ plots of aggregate (A) total cell count, (B) % neuron-PI co-localization, (C) % cell damage, (D) % microglia-PI co-localization and (E) microglia-DAPI co-localization for all relevant experimental groups.

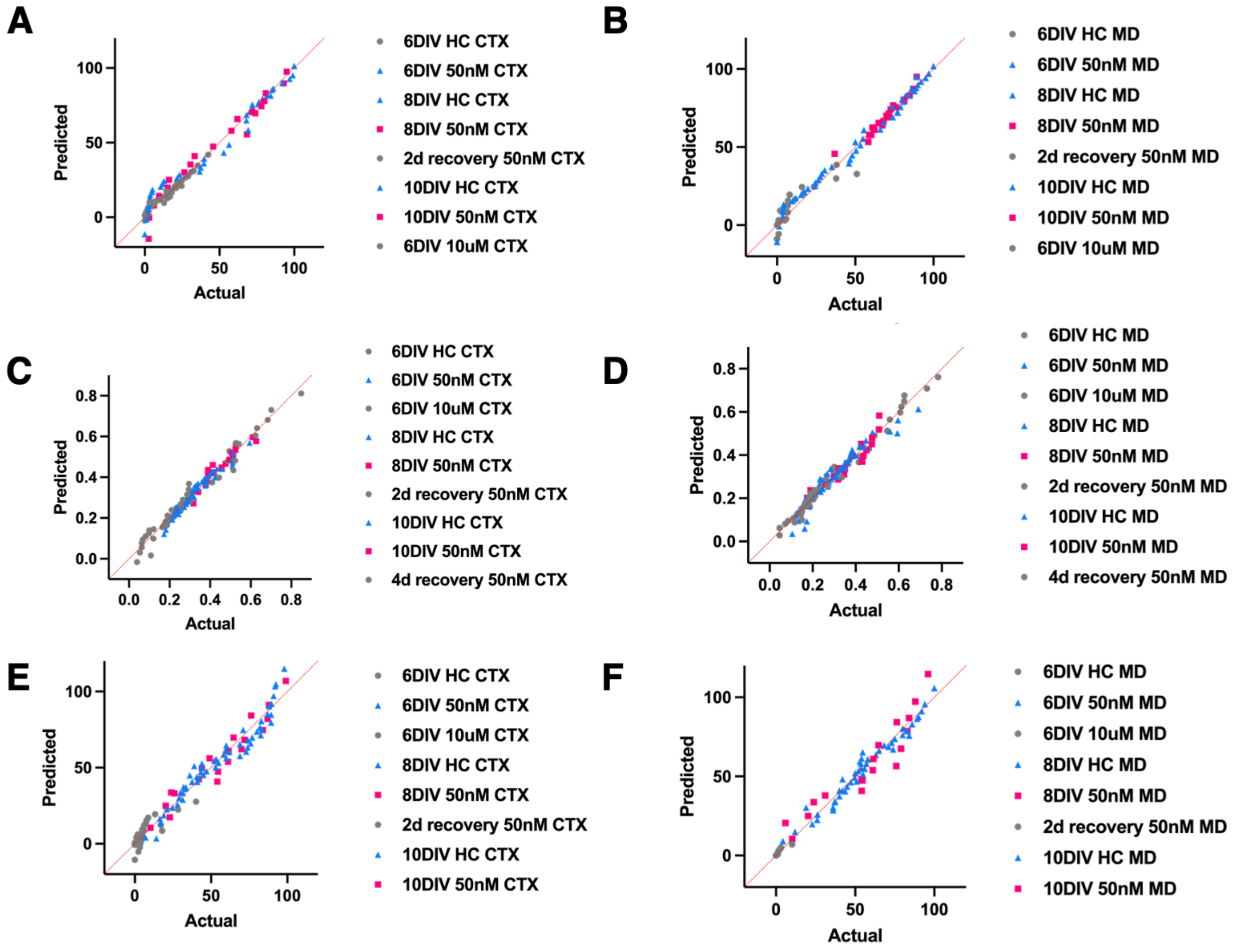

Figure S2. Normality QQ plots of regional % NeuN-PI co-localization in the **(A)** cortex and **(B)** midbrain, % microglia-DAPI co-localization (density) in the **(C)** cortex and **(D)** midbrain, **%** microglia-PI co-localization in the **(E)** cortex and **(F)** midbrain for all relevant experimental groups.

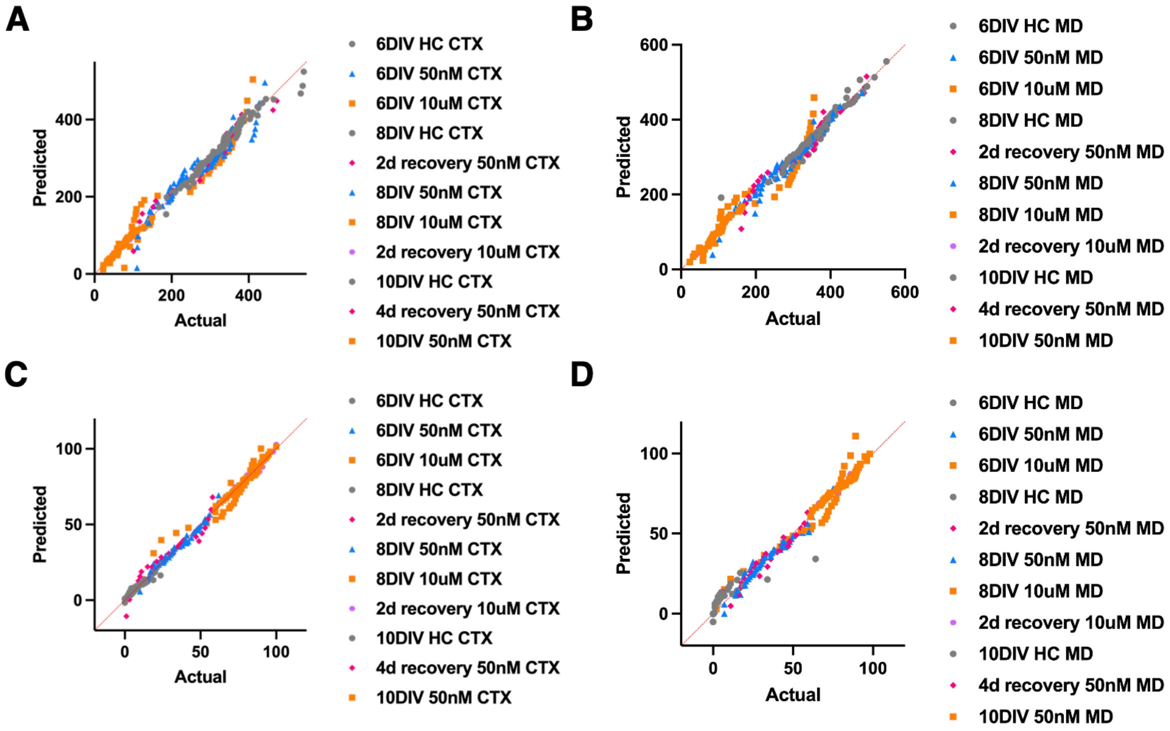

Figure S3. Normality QQ plots of regional cell counts in the **(A)** cortex and **(B)** midbrain and % cell damage in the **(C)** cortex and **(D)** midbrain for all relevant experimental groups.

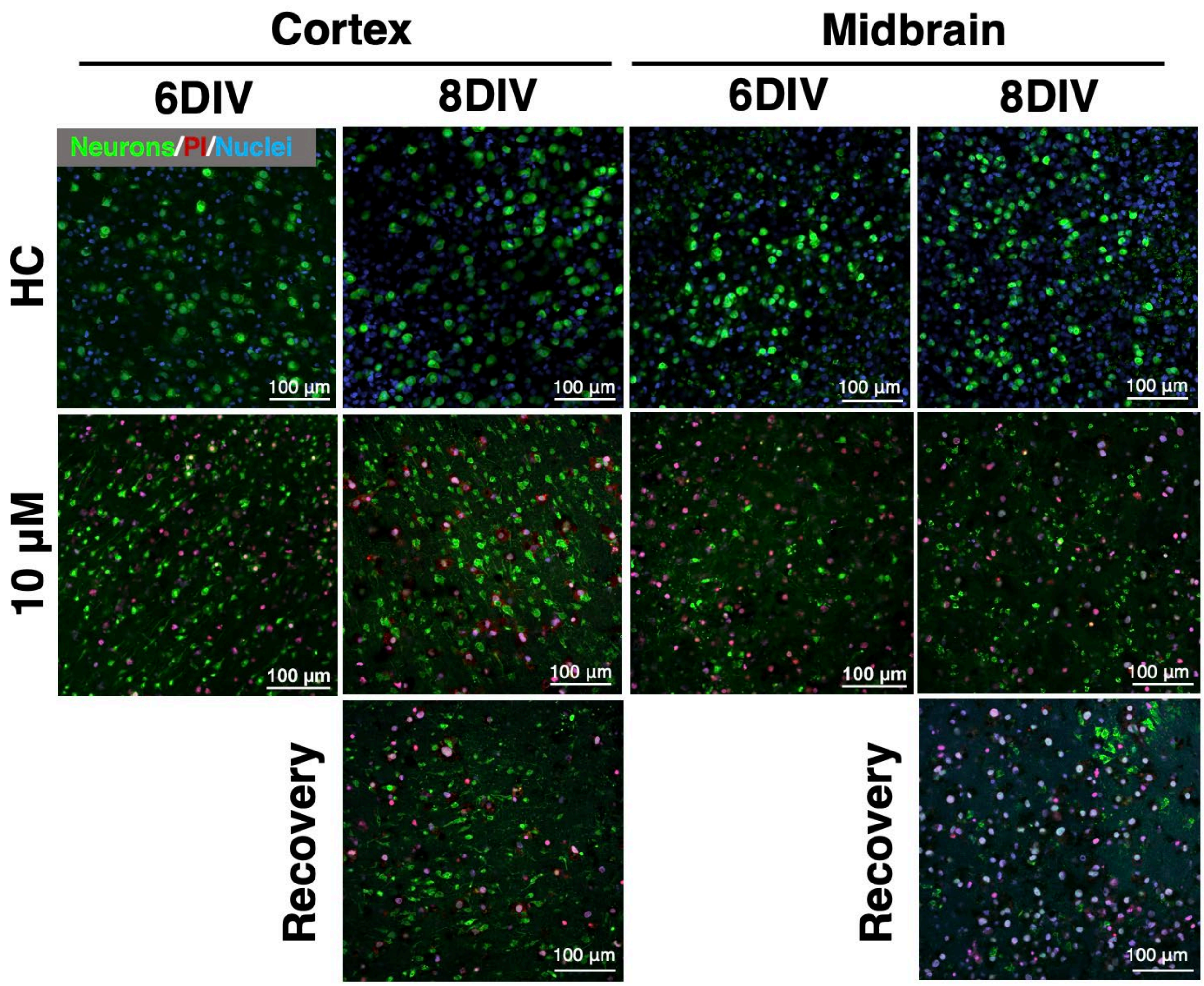

Figure S4. Representative images of NeuN+ neurons in the cortex and midbrain of OWH slices (N=2 OWH slices/condition) exposed to 10 μM ROT with n=5-10 images taken at 40x magnification per condition in each region).

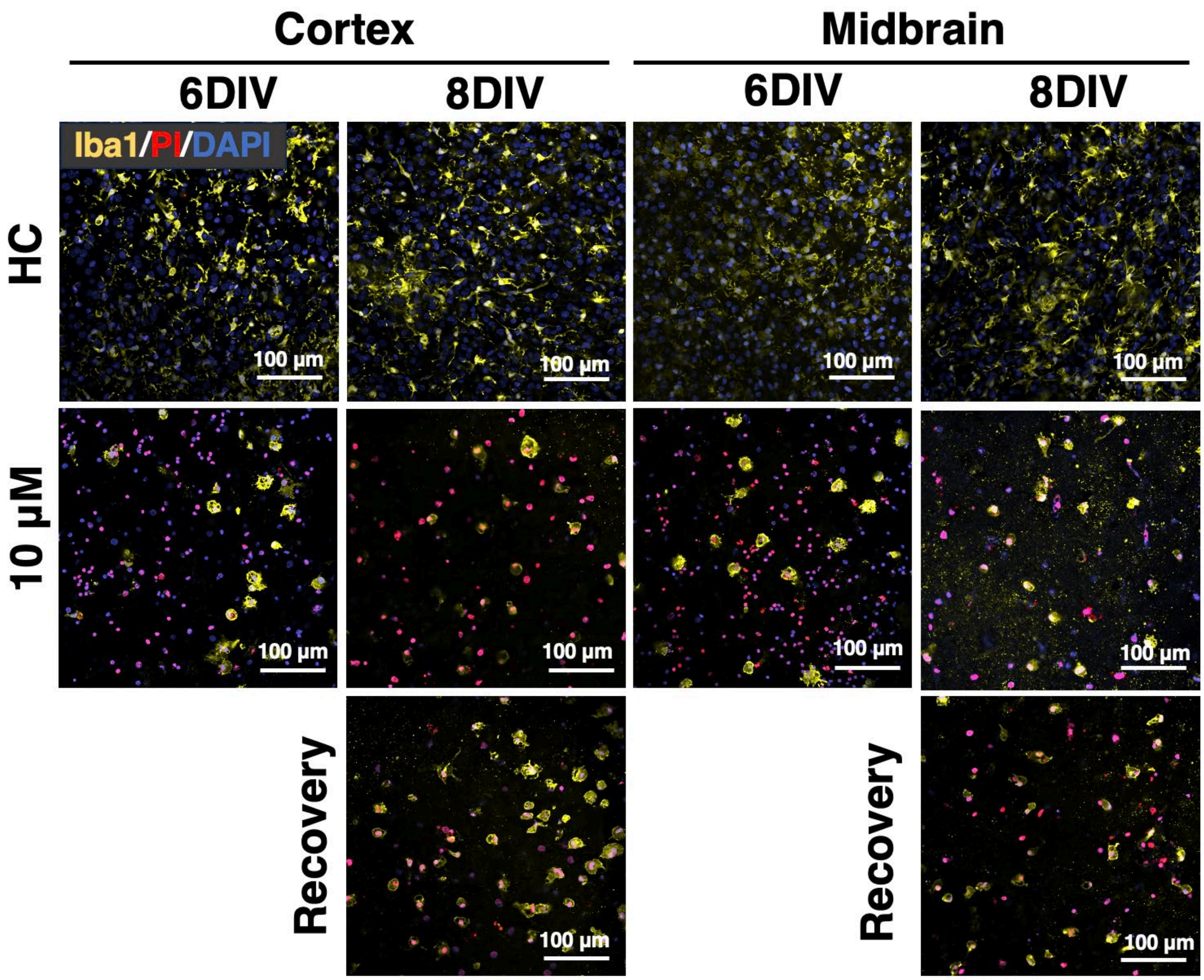

Figure S5. Representative images of Iba1+ microglia in the cortex and midbrain of OWH slices (N=2 OWH slices/condition) exposed to 10 μM ROT with n=5-10 images taken at 40x magnification per condition in each region).

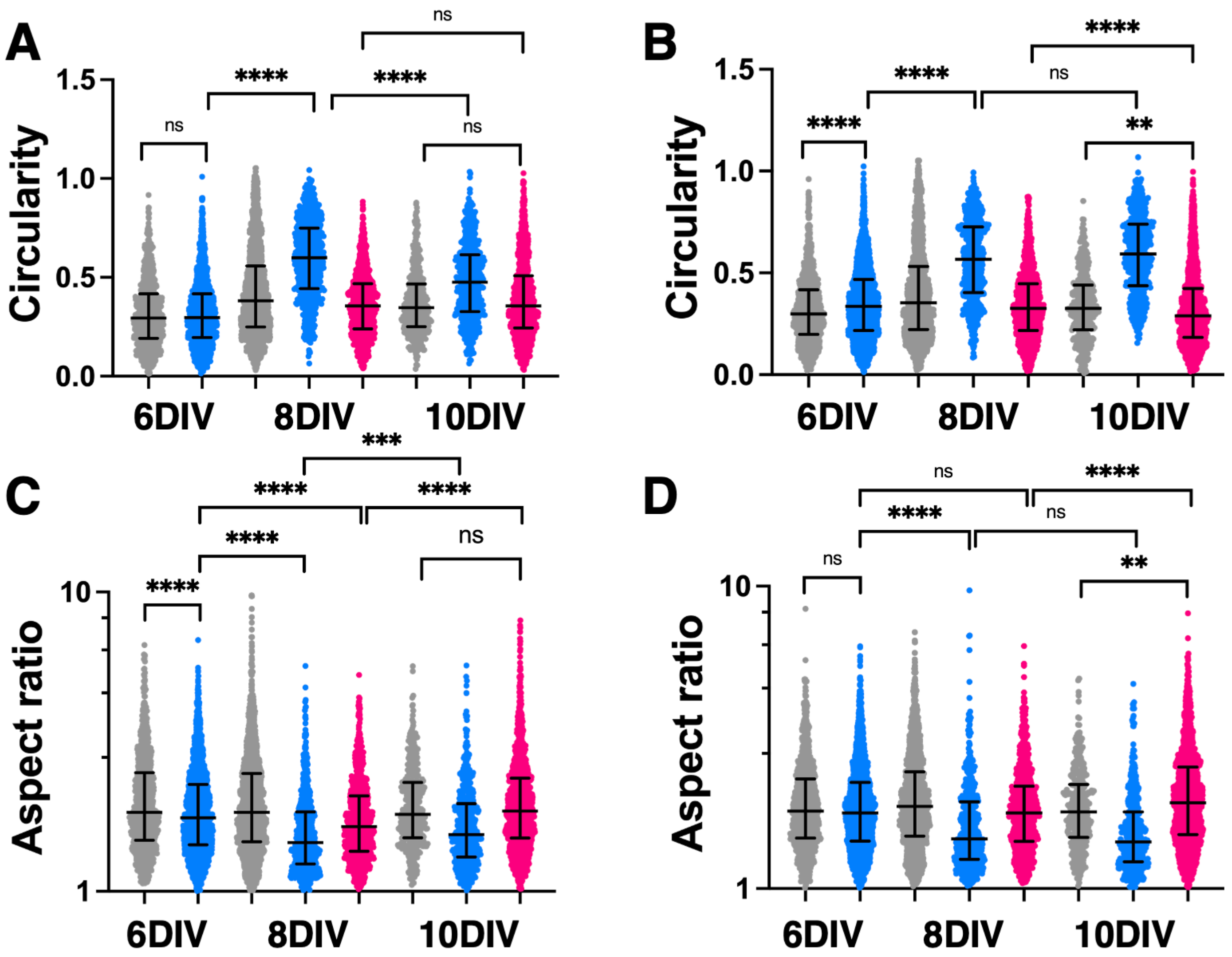

Figure S6. Regional differences across experimental groups for microglial morphological features. Circularity (4π*area/perimeter^2) of Li-thresholded microglia in the **(A)** cortex and **(B)** midbrain of OWH slices. Aspect ratio (major axis length/minor axis length) of Li-thresholded microglia in the **(C)** cortex and **(D)** midbrain of OWH slices. Each datapoint represents a single cell in an image (N=2-3 OWH slices/ group/region) with n=5-10 images taken at 40x magnification. All code available at https://github.com/Nance-Lab/cellmorphflows.

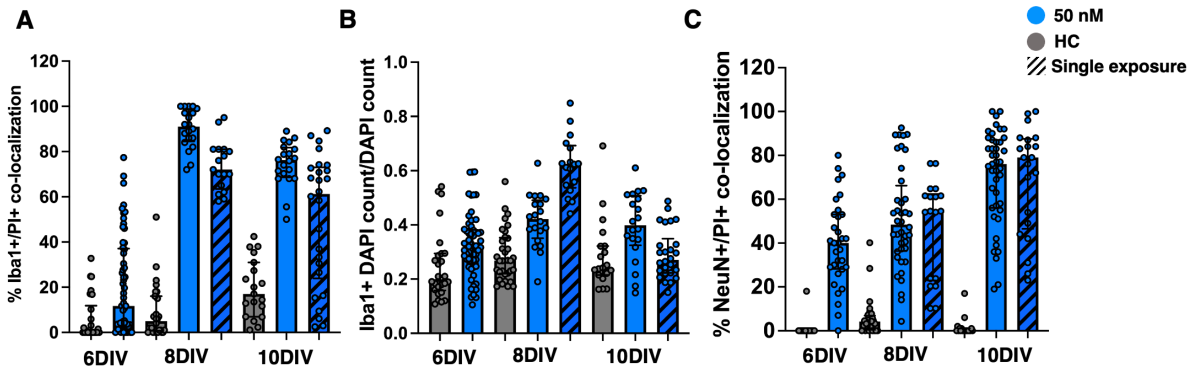

Figure S7. Aggregate (A) % microglia-PI+ co-localization, (B) % microglia-DAPI+ co-localization and (C) % neuron-PI+ co-localization damage, (D) % microglia-PI co-localization and for OWH slices (N=2-3 OWH slices/group) from n=5-10 images taken at 40x magnification per condition in each region.

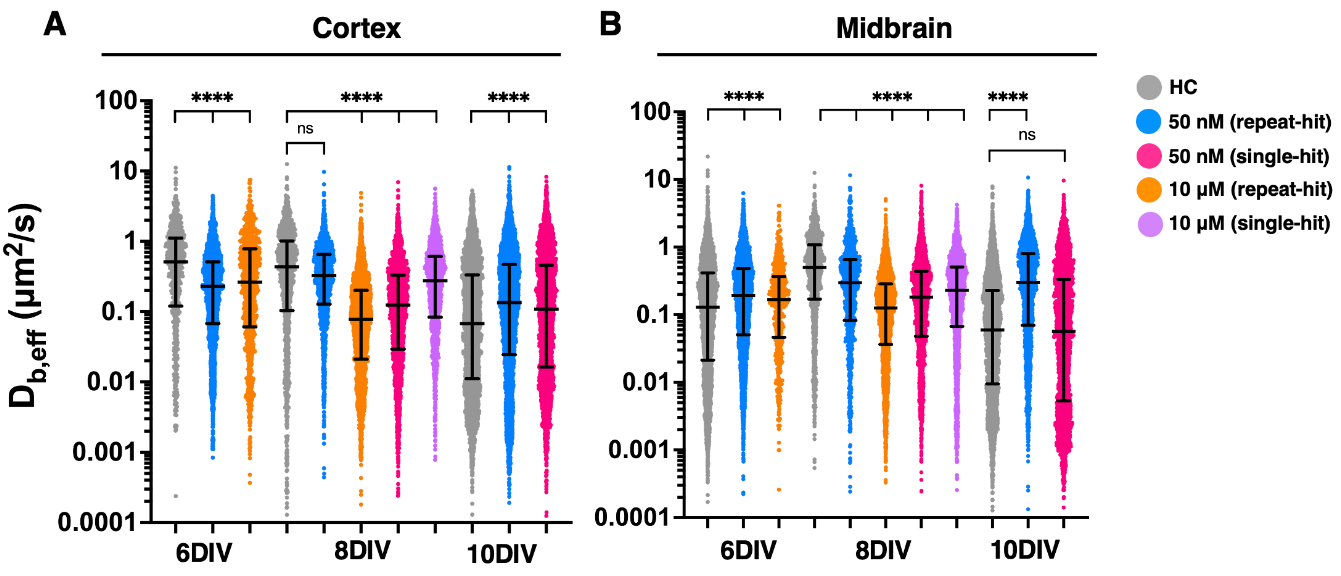

Figure S8. Distributions of effective diffusion coefficients ($D_{b,eff}$) comparing 40 nm PS-PEG particle diffusion for all experimental groups in the **(A)** cortex and **(B)** midbrain. Particles were tracked in 2-3 slices per each condition, with 3-5 videos were collected in each region per condition, resulting in > 2,000 trajectories per condition. All videos were acquired at 67 frames per second, 100x magnification, and within 1 hour following particle incubation. Representative effective diffusion coefficients and viscosities are calculated at a trajectory lag time of $\tau = 0.1$ s. For grouped significance bars, number of asterisks (*) denotes significance relative to healthy (gray) distributions.
